# Welcoming care practices for migrant children in Primary Health Care: a scoping review

**DOI:** 10.1590/1980-220X-REEUSP-2026-0121en

**Published:** 2026-08-31

**Authors:** Ana Beatriz Oliveira Costa, Lanna Jeniffer Silva Rodrigues, Mônica Letícia Martins Franco, Paulo Sérgio da Silva, Audrey Vidal Pereira, Liliane Faria da Silva

**Affiliations:** 1 Universidade Federal Fluminense, Programa Acadêmico em Ciências do Cuidado em Saúde, Niterói, RJ, Brazil.; 2 Universidade Federal de Lavras, Faculdade de Ciências da Saúde, Lavras, MG, Brazil.

**Keywords:** User Embracement, Primary Health Care, Comprehensive Health Care, Transients and Migrants

## Abstract

**Objective:**

To map and synthesize the available evidence on welcoming care practices directed toward migrant children in Primary Health Care.

**Method:**

A scoping review conducted according to the recommendations of the JBI and reported in accordance with the PRISMA-ScR checklist. Searches were performed in the MEDLINE/PubMed, CINAHL, Academic Search Premier, Academic Search Ultimate (EBSCO), Scopus, EMBASE/Elsevier, APA PsycInfo (VHL), LILACS, BDENF, Index Psychology – Periodicals, and SciELO databases.

**Results:**

Forty-two studies published between 1999 and 2024 were included. The evidence was categorized into clinical and follow-up actions in Primary Health Care, preventive health actions in Primary Health Care, and healthcare network coordination. The included studies showed heterogeneity regarding methodological designs, study settings, and the characteristics of the populations investigated.

**Conclusion:**

Welcoming care has been concentrated on clinical, preventive, and healthcare network coordination actions, reaffirming Primary Health Care as the main entry point for child healthcare. However, the findings reveal the need for future studies addressing culturally competent welcoming strategies, structured intersectoral interventions, and the assessment of the effectiveness of these practices.

## INTRODUCTION

Initially, it is important to contextualize that migration consists of the movement of populations between different regions. According to its spatial scope, migration may be classified as internal, when restricted to the territorial boundaries of a sovereign state, or international, when it involves crossing national borders and settling in a foreign country^(1)^. Indeed, migration directly influences migrants’ health, and the process of adapting to a new location is not always positive. Migrants may become vulnerable because of their social condition and, in some cases, experience isolation and separation from their families, which increases the likelihood of stigma, discrimination, and prejudice^(2)^.

Upon entering a new country, migrants encounter barriers to accessing healthcare services, including the absence of health insurance, fear of deportation, exclusion from government programs, and linguistic and cultural differences^(3)^. Specifically, children in situations of mobility experience high social vulnerability and are exposed to severe risks, such as food insecurity, human trafficking, sexual exploitation, and neglect of healthcare. In this regard, the migration process, which involves displacement and crossing borders under conditions of repression, in addition to the complex process of sociocultural readaptation, exposes children to stressful events with profound and long-lasting repercussions for their mental health^(4)^.

Given these considerations and the specific characteristics of the migrant child population, Primary Health Care (PHC) is a central strategy in public health, particularly because it longitudinally monitors children’s growth and development, encourages community participation in healthcare services, and contributes to promoting the well-being of populations. Furthermore, it is important to note that PHC has contributed to strengthening the relationships between the community and the actors within the Unified Health System (SUS - *Sistema* Único *de Saúde*), thereby fostering empowerment at the local and regional levels^(5)^.

Within this perspective, the health needs of the migrant child population in PHC can be recognized through user embracement, understood as an approach that recognizes users and their health needs as legitimate and unique, while sustaining the relational and human dimensions of the care process. The act of welcoming may involve other care tools, such as qualified listening, which contributes to building bonds and achieving a deeper understanding of migration-related needs^(6)^.

In Brazil, over the years, child health has advanced in the organization of care within PHC. This improvement in the quality of care is the result of the consolidation of children’s legal rights and the evolution of public health policies in the national context. In PHC, children receive healthcare based on the principles of the universal right to life, equity, comprehensive care, humanization, care, and participatory management^(7)^.

Based on these preliminary considerations, a preliminary search was conducted in April 2024 in the International Prospective Register of Systematic Reviews (PROSPERO) and the main databases. No published or ongoing scoping or systematic review addressing the specific characteristics of the migrant child population was identified. Within the context presented, little has been discussed in detail regarding welcoming care practices for migrant children provided by healthcare professionals in PHC.

From this perspective, a scoping review was conducted with the objective of mapping and synthesizing the available evidence on welcoming care practices directed toward migrant children in PHC. Based on these objectives, the review sought to identify knowledge gaps, promote equity in access to healthcare, support public policies, and improve the quality of care provided to migrant children in PHC, thereby ensuring culturally sensitive care that meets the needs of the migrant child population.

## METHOD

### Study Design

This is a scoping review developed in accordance with the methodological guidance provided by the JBI^(8)^. This type of review aims to map the main scientific evidence and the limitations of a given topic available in the literature and was conducted according to the recommendations of the Preferred Reporting Items for Systematic Reviews and Meta-Analyses extension for Scoping Reviews (PRISMA-ScR)^(9)^. During the exploratory phase of the review, the objective was to verify the absence of recent research reports or review protocols addressing a topic similar to that of the present study. Subsequently, the review protocol was registered on the Open Science Framework platform (https://doi.org/10.17605/OSF.IO/ZSTNX).

Considering that this is a scoping review, whose purpose is to map and characterize the evidence available in the literature, no formal critical appraisal of the methodological quality of the included studies was performed. This decision is consistent with the JBI recommendations, which do not require quality appraisal in scoping reviews, especially when the focus is on the breadth of the evidence and the identification of knowledge gaps.

### Identification of the Research Question

The Population, Concept, and Context (PCC) framework was adopted to formulate the research question. This strategy assists in identifying the key topics and is recommended for conducting scoping reviews. Thus, this study was guided by the following research question: What are the welcoming practices for migrant children aged 0 to 10 years in PHC?

Studies involving children aged 0 to 10 years as the target population, with a specific focus on migrants, were included. In this study, migrants are defined as individuals who move from their usual place of residence, either within a country or across an international border, on a temporary or permanent basis^(10)^.

### Selection Criteria

This scoping review included experimental and quasi-experimental studies, including randomized clinical trials, non-randomized clinical trials, before-and-after studies, and interrupted time-series studies. Analytical observational studies, including prospective and retrospective cohort studies, case-control studies, and cross-sectional studies, were also included. Descriptive observational studies, such as case series, individual case reports, and descriptive cross-sectional studies, were also considered. Qualitative studies using different approaches, including phenomenology, grounded theory, ethnography, qualitative description, action research, and feminist research, were included. Reviews of any type that met the inclusion criteria were considered in accordance with the research question. Finally, texts and opinion articles were also included in this scoping review. Abstracts and letters to the editor were excluded.

Considering that this is a scoping review, whose purpose is to map and characterize the evidence available in the literature, no formal critical appraisal of the methodological quality of the included studies was performed. This decision is consistent with JBI recommendations, which do not require quality appraisal in scoping reviews, especially when the focus is on the breadth of the evidence and the identification of knowledge gaps.

### Search Strategy

The search strategy was conducted to identify both published and unpublished studies. Initially, the preliminary search was performed using terms from the Health Sciences Descriptors, the Medical Subject Headings, and the Embase Subject Headings, which were combined using the Boolean operators OR and AND in MEDLINE/PubMed, OSF, PROSPERO, JBI Synthesis, and the Cochrane Library. The objective was to identify protocols, reviews, and primary studies, as well as new keywords and standardized indexing terms for a comprehensive search strategy.

Subsequently, the identified terms were used to develop the final search strategy, which was adapted to the databases and information sources selected for the review, namely: MEDLINE/PubMed; Cumulative Index to Nursing and Allied Health Literature (CINAHL); Academic Search Premier and Academic Search Ultimate (EBSCO); Scopus; EMBASE/Elsevier; and APA PsycInfo. The databases of the Regional Portal of the Virtual Health Library (VHL) were also searched, including *Literatura Latino-Americana e do Caribe em Ciências da Saúde* (LILACS), *Base de Dados de Enfermagem* (BDENF), and Index Psychology – Periodicals, as well as the Scientific Electronic Library Online (SciELO). Studies were included regardless of language or publication date.

Chart 1 presents the search strategies adopted for each database, organized according to the PCC framework, including descriptors and keywords arranged by block, the Boolean logic applied, and the number of records retrieved.

**Chart 1:** Search strategy conducted on May 10, 2024, for each database – Niterói, RJ, Brazil, 2024.

Database	Search strategy	Records found (n)
LILACSPLUS/VHL	Migrantes OR Migrante OR Nômade OR Nômades OR Nomadismo OR Não-migrante OR Não-migrantes OR Posseiro OR Posseiros OR Transeunte OR Transeuntes OR Nómada OR Nómadas OR Nomadismo OR Ocupante OR Migratorio OR Population de Passage et Migrants OR Migrants OR Nomade OR Population de passage OR Population en Transit OR Population en Transit et Migrants OR Sédentaires OR Squatters OR Squatteurs OR Squatteuses OR Transients and Migrants OR Migrant OR Nomad OR Nonmigrant OR Squatter OR Transien; AND Criança OR Infantil OR Infancia OR Infant, Newborn OR Newborn Infant OR Newborn OR Neonate OR Infant OR Child, Preschool OR Preschool OR Full Term Infant OR Neonatus OR Newly Born Baby OR Newly Born Child OR Newly Born Infant OR Pediatric OR Infant, Premature OR Preterm OR Premature OR Neonatal Prematurity OR Prematurita OR Recem-Nascido OR Neonato OR Lactente OR Prematuro OR Infancia OR Cuidado do Lactente OR Cuidado del Lactante OR Atención del Lactante OR Soins du Nourrisson OR Soins au Nouveau-né et au Nourrisson OR Soins aux Nouveau-nés et aux Nourrissons OR Soins des Nourrissons OR Soins Infantiles OR Cuidado da Criança OR Cuidado Infantil OR puericultura OR Cuidado del Niño OR Cuidado Infantil OR Puericultura OR Soins de L’enfant OR Soins aux Enfants OR Soins des Enfants OR Infant Care OR Child Care OR Child Day Care OR puericulture; AND Acolhimento OR User Embracement OR Acogimiento OR Adoption par l’Utilisateur OR Amparo OR Assistência OR Abrigamento OR Cuidado OR Support OR Care OR Welcoming OR Apoyo OR Asistencia OR Bienvenida OR Integración OR Adoption par l’Utilisateur OR Accueil OR Soutien OR Assistance OR Bienvenue OR Intégration; OR Criança Acolhida OR Criança Bem-Vinda OR Criança em Regime de Acolhimento OR Crianças Acolhidas OR Crianças Bem-Vindas OR Crianças em Regime de Acolhimento OR Jovem Acolhido OR Jovem Bem-Vindo OR Jovens Acolhidos OR Jovens Bem-Vindos OR Niño Acogido OR Joven Acogido OR Jóvenes en Régimen de Acogida OR Niña Acogida OR Niños Acogidos OR Enfant Placé en Famille d’accueil OR Enfant Accueilli OR Enfants accueillis OR Enfants Placés en Familles D’accueil OR Jeune Accueilli OR Jeune Placé en Famille D’accueil OR Child, Foster OR Foster Child OR Foster Children OR Foster Youth OR Foster Youths; AND Atenção Primária à Saúde OR Atención Primaria de Salud OR Atendimento Básico OR Atendimento Primário OR Atenção Básica OR Atenção Básica de Saúde OR Atenção Básica à Saúde OR Atenção Primária OR Atenção Primária de Saúde OR Atenção Primária em Saúde OR Cuidados Primários OR Cuidados Primários de Saúde OR Cuidados Primários à Saúde OR Cuidados de Saúde Primários OR Centros de Saúde OR Centros de Salud OR Centro de Saúde OR Posto de Assistência Médica OR Posto de Saúde OR Postos de Saúde OR Unidade Básica de Saúde OR Serviços Básicos de Saúde OR Servicios Básicos de Salud OR Prestações Básicas de Saúde OR Unidade de Saúde OR Unidade de Serviço OR Prestaciones Básicas de Salud OR Centro de Salud OR Postas Médicas OR Puestos Médicos OR Puestos de Salud OR Primary Health Care OR Primary Healthcare OR Primary Care OR Health Centers OR Health Center OR Health Posts OR Basic Health Services OR Basic Health Care Provisions OR Community Health Services OR Community Health Care OR Community Health Service OR Community Healthcare OR Assistência à Saúde Comunitária OR Assistência Médica Comunitária OR Cuidados Médicos Comunitários OR Atención de la Salud Comunitaria OR Atención Médica Comunitaria OR Services de Santé Communautaires OR Services de Santé Communautaire OR Services de Santé de Proximité OR Soins de Santé Communautaires OR Soins de Santé de Proximité; AND Instance: lilacsplus.	52
CINAHL and SocINDEX/EBSCO	Puericulture OR Childcare OR Infant, Newborn OR Newborn Infant OR Newborn OR Neonate OR Infant OR Infant OR Child, Preschool OR Preschool OR Full Term Infant OR Neonatus OR Newly Born Baby OR Newly Born Child OR Newly Born Infant OR Pediatric OR Infant, Premature OR Preterm OR Premature OR Neonatal Prematurity OR prematurita OR Child OR Child OR Children; AND User Embracement OR support OR care OR welcoming OR Embracement; OR Child, Foster OR Foster Child OR Foster Youth OR Infant Care OR Child Care OR Child Day Care; OR Puericulture OR Childcare OR Infant, Newborn OR Newborn Infant OR Newborn OR Neonate OR Infant OR Infant OR Child, Preschool OR Preschool OR Full Term Infant OR Neonatus OR Newly Born Baby OR Newly Born Child OR Newly Born Infant OR Pediatric OR Infant, Premature OR Preterm OR Premature OR Neonatal Prematurity OR Prematurita OR Child OR Child OR Children AND User Embracement OR Support OR Care OR Welcoming OR Embracement OR Child, Foster OR Foster Child OR Foster Youth OR Infant Care OR Child Care OR Child Day Care OR; Puericulture OR childcare OR Infant, Newborn OR Newborn Infant OR Newborn OR Neonate OR Infant OR Infant OR Child, Preschool OR Preschool OR Full Term Infant OR Neonatus OR Newly Born Baby OR Newly Born Child OR Newly Born Infant OR Pediatric OR Infant, Premature OR Preterm OR Premature OR Neonatal Prematurity OR Prematurita OR Child OR Child OR Children AND User Embracement OR Support OR Care OR Welcoming OR Embracement; OR Child, Foster OR Foster Child OR Foster Youth OR Infant Care OR Child Care OR Child Day Care; AND Transients and Migrants OR Migrant OR Nomad OR Nonmigrant OR Squatter OR Transient OR Refugee OR Expatriate OR Immigrant OR Transients and Migrants OR Migrant OR Nomad OR Nonmigrant OR Squatter OR Transient OR Refugee OR Expatriate OR Immigrant OR Transients and Migrants OR Migrant OR Nomad OR Nonmigrant OR Squatter OR Transient OR Refugee OR Expatriate OR Immigrant; AND Primary Health Care OR Primary Healthcare OR Primary Care OR Health Centers OR Health Center OR Health Posts OR Polyclinic OR Community Health Services OR Community Health Care OR Community Health Service OR Community Healthcare OR Primary Health Care OR Primary Healthcare OR Primary Care OR Health Centers OR Health Center OR Health Posts OR Polyclinic OR Community Health Services OR Community Health Care OR Community Health Service OR Community Healthcare OR Primary Health Care OR Primary Healthcare OR Primary Care OR Health Centers OR Health Center OR Health Posts OR Polyclinic OR Community Health Services OR Community Health Care OR Community Health Service OR Community Healthcare.	517
EMBASE/Elsevier	Puericulture:ti,ab,kw OR Childcare:ti,ab,kw OR Infant, Newborn’:ti,ab,kw OR Newborn Infant*’:ti,ab,kw OR Newborn*:ti,ab,kw OR Neonate*:ti,ab,kw OR ‘Infant’:ti,ab,kw OR Infant*:ti,ab,kw OR ‘Child, Preschool’:ti,ab,kw OR Preschool*:ti,ab,kw OR ‘Full term Infant’:ti,ab,kw OR Neonatus:ti,ab,kw OR ‘Newly Born Baby’:ti,ab,kw OR ‘Newly Born Child’:ti,ab,kw OR ‘Newly Born Infant’:ti,ab,kw OR Pediatric*:ti,ab,kw OR ‘Infant, Premature’:ti,ab,kw OR Preterm*:ti,ab,kw OR Premature*:ti,ab,kw OR ‘Neonatal Prematurity’:ti,ab,kw OR Prematurita*:ti,ab,kw OR ‘Child’:ti,ab,kw OR Child*:ti,ab,kw OR Children:ti,ab,kw) AND; ‘User Embracement’:ti,ab,kw OR Support:ti,ab,kw OR Care:ti,ab,kw OR Welcoming:ti,ab,kw OR Embracement:ti,ab,kw; OR ‘Child, Foster’:ti,ab,kw OR ‘Foster Child*’:ti,ab,kw OR ‘Foster Youth*’:ti,ab,kw OR ‘Infant Care’:ti,ab,kw OR ‘Child Care’:ti,ab,kw OR ‘Child Day Care’:ti,ab,kw; AND Transients:ti,ab,kw AND Migrants:ti,ab,kw OR Migrant*:ti,ab,kw OR Nomad*:ti,ab,kw OR Nonmigrant*:ti,ab,kw OR Squatter*:ti,ab,kw OR Transient*:ti,ab,kw OR Refugee*:ti,ab,kw OR Expatriate*:ti,ab,kw OR Immigrant*:ti,ab,kw; AND ‘Primary Health Care’:ti,ab,kw OR ‘Primary Healthcare’:ti,ab,kw OR ‘Primary Care’:ti,ab,kw OR ‘Health Centers’:ti,ab,kw OR ‘Health Center’:ti,ab,kw OR ‘Health Posts’:ti,ab,kw OR Polyclinic:ti,ab,kw OR ‘Community Health Services’:ti,ab,kw OR ‘Community Health Care’:ti,ab,kw OR ‘Community Health Service’:ti,ab,kw OR ‘Community Healthcare’:ti,ab,kw; AND [article]/lim OR [article in press]/lim OR [conference review]/lim OR [review]/lim OR [preprint]/lim.	568
PMC/NLM	Puericulture OR Childcare OR Infant, Newborn OR Newborn Infant OR Newborn OR Neonate OR Infant OR Infant OR Child, Preschool OR Preschool OR Full Term Infant OR Neonatus OR Newly Born Baby OR Newly Born Child OR Newly Born Infant OR Pediatric OR Infant, Premature OR Preterm OR Premature OR Neonatal Prematurity OR Prematurita OR Child OR Children AND User Embracement OR Support OR Care OR Welcoming OR Embracement OR Child, Foster OR Foster Child OR Foster Youth OR Infant Care OR Child Care OR Child Day Care AND Transients AND Migrants OR Migrant OR Nomad OR Nonmigrant OR Squatter OR Transient OR Refugee OR Expatriate OR Immigrant AND Primary Health Care OR Primary Healthcare OR Primary Care OR Health Centers OR Health Center OR Health Posts OR Polyclinic OR Community Health Services OR Community Health Care OR Community Health Service OR Community Healthcare.	473
PsycInfo/APA	Primary Health Care OR Primary Healthcare OR Primary Care OR Health Centers OR Health Center OR Health Posts OR Polyclinic OR Community Health Services OR Community Health Care OR Community Health Service OR Community Healthcare OR Primary Health Care OR Primary Healthcare OR Primary Care OR Health Centers OR Health Center OR Health Posts OR Polyclinic OR Community Health Services OR Community Health Care OR Community Health Service OR Community Healthcare; Primary Health Care OR Primary Healthcare OR Primary Care OR Health Centers OR Health Center OR Health Posts OR Polyclinic OR Community Health Services OR Community Health Care OR Community Health Service OR Community Healthcare AND Transients and Migrants OR Migrant OR Nomad OR Nonmigrant OR Squatter OR Transient OR Refugee OR Expatriate OR Immigrant OR Transients and Migrants OR Migrant OR Nomad OR Nonmigrant OR Squatter OR Transient OR Refugee OR Expatriate OR Immigrant OR Transients and Migrants OR Migrant OR Nomad OR Nonmigrant OR Squatter OR Transient OR Refugee OR Expatriate OR Immigrant AND Puericulture OR Childcare OR Infant, Newborn OR Newborn Infant OR Newborn OR Neonate OR Infant OR Infant OR Child, Preschool OR Preschool OR Full term Infant OR Neonatus OR Newly Born Baby OR Newly Born Child OR Newly Born Infant OR Pediatric OR Infant, Premature OR Preterm OR Premature OR Neonatal Prematurity OR Prematurita OR Child OR Child OR Children AND User Embracement OR Support OR Care OR Welcoming OR Embracement OR Child, Foster OR Foster Child OR Foster Youth OR Infant Care OR Child Care OR Child Day Care OR Puericulture OR Childcare OR Infant, Newborn OR Newborn Infant OR Newborn OR Neonate OR Infant OR Infant OR Child, Preschool OR Preschool OR Full Term Infant OR Neonatus OR Newly Born Baby OR Newly Born Child OR Newly Born Infant OR Pediatric OR Infant, Premature OR Preterm OR Premature OR Neonatal Prematurity OR Prematurita OR Child OR Child OR Children AND User Embracement OR Support OR Care OR Welcoming OR Embracement OR Child, Foster OR Foster Child OR Foster Youth OR Infant Care OR Child Care OR Child Day Care OR Puericulture OR Childcare OR Infant, Newborn OR Newborn Infant OR Newborn OR Neonate OR Infant OR Infant OR Child, Preschool OR Preschool OR Full Term Infant OR Neonatus OR Newly Born Baby OR Newly Born Child OR Newly Born Infant OR Pediatric OR Infant, Premature OR Preterm OR Premature OR Neonatal Prematurity OR Prematurita OR Child OR Children AND User Embracement OR Support OR Care OR Welcoming OR Embracement OR Child, Foster OR Foster Child OR Foster Youth OR Infant Care OR Child Care OR Child Day Care.	176
PubMed/NLM	Puericulture OR Childcare OR Infant, Newborn OR Newborn Infant OR Newborn OR Neonate OR Infant OR Infant OR Child, Preschool OR Preschool OR Full Term Infant OR Neonatus OR Newly Born Baby OR Newly Born Child OR Newly Born Infant OR Pediatric OR Infant, Premature OR Preterm OR Premature OR Neonatal Prematurity OR prematurita OR Child OR Children AND Embracement OR Support OR Care OR Welcoming OR Embracement OR Child, Foster OR Foster Child OR Foster Youth OR Infant Care OR Infant Care OR Child Care OR Child Day Care AND Transients and Migrants OR Transients and Migrants OR Migrant OR Nomad OR Nonmigrant OR Squatter OR Transient OR Refugee OR Expatriate OR Immigrant AND Primary Health Care OR Primary Health Care OR Primary Healthcare OR Primary Care OR Health Centers OR Health Center OR Health Posts OR Polyclinic OR Community Health Services OR Community Health Care OR Community Health Service OR Community Healthcare.	1,431
SciELO	Puericulture OR Childcare OR Infant, Newborn OR Newborn Infant OR Newborn OR Neonate OR Infant OR Infant OR Child, Preschool OR Preschool OR Full Term Infant OR Neonatus OR Newly Born Baby OR Newly Born Child OR Newly Born Infant OR Pediatric OR Infant, Premature OR Preterm OR Premature OR Neonatal Prematurity OR prematurita OR Child OR Children AND User Embracement OR Support OR Care OR Welcoming OR Embracement OR Child, Foster OR Foster Child OR Foster Youth OR Infant Care OR Child Care OR Child Day Care AND Transients and Migrants OR Migrant OR Nomad OR Nonmigrant OR Squatter OR Transient OR Refugee OR Expatriate OR Immigrant AND Primary Health Care OR Primary Healthcare OR Primary Care OR Health Centers OR Health Center OR Health Posts OR Polyclinic OR Community Health Services OR Community Health Care OR Community Health Service OR Community Healthcare.	28
Scopus/Elsevier	Puericulture OR Childcare OR Infant, Newborn OR Newborn Infant OR Newborn OR Neonate OR Infant OR Infant OR Child, Preschool OR Preschool OR Full Term Infant OR Neonatus OR Newly Born Baby OR Newly Born Child OR Newly Born Infant OR Pediatric OR Infant, Premature OR Preterm OR Premature OR Neonatal Prematurity OR Prematurita OR Child OR Children AND User Embracement OR Support OR Care OR Welcoming OR Embracement OR Child, Foster OR Foster Child OR Foster Youth OR Infant Care OR Child Care OR Child Day Care AND Transients and Migrants OR Migrant OR Nomad OR Nonmigrant OR Squatter OR Transient OR Refugee OR Expatriate OR Immigrant AND Primary Health Care OR Primary Healthcare OR Primary Care OR Health Centers OR Health Center OR Health Posts OR Polyclinic OR Community Health Services OR Community Health Care OR Community Health Service OR Community Healthcare.	1,457
Web of Science/Clarivate	Puericulture OR Childcare OR Infant, Newborn OR Newborn Infant OR Newborn OR Neonate OR Infant OR Infant OR Child, Preschool OR Preschool OR Full Term Infant OR Neonates OR Newly Born Baby OR Newly Born Child OR Newly Born Infant OR Pediatric OR Infant, Premature OR Preterm OR Premature OR Neonatal Prematurity OR Prematurita OR Child OR Child OR Children AND User Embracement OR Support OR Care OR Welcoming OR Embracement OR Child, Foster OR Foster Child OR Foster Youth OR Infant Care OR Child Care OR Child Day Care AND Transients and Migrants OR Migrant OR Nomad OR Nonmigrant OR Squatter OR Transient OR Refugee OR Expatriate OR Immigrant AND Primary Health Care OR Primary Healthcare OR Primary Care OR Health Centers OR Health Center OR Health Posts OR Polyclinic OR Community Health Services OR Community Health Care OR Community Health Service OR Community Healthcare.	687

As search strategies listed in the schematic chart were adapted to the functional characteristics of each database, considering their respective indexing fields, the application of Boolean operators, and the specific information retrieval resources. To broaden the findings of this study, the reviewers developed the search strategy with the assistance of a librarian experienced in scoping reviews, in order to ensure the comprehensiveness, sensitivity, and appropriateness of the descriptors and keywords used. The strategies were validated and adapted to the specific characteristics of each database, respecting their indexing mechanisms and information retrieval processes.

### Study Selection

The studies retrieved from the database searches were imported into EndNote 21 (Clarivate Analytics, PA, USA), and duplicate records were removed. The references were then exported to the Intelligent Systematic Review (Rayyan) software for selection management. In the first phase, the studies were assessed based on their titles and abstracts according to the inclusion criteria, and in the second phase, the full texts of the potentially eligible studies were retrieved and assessed in their entirety. In the third and final phase, the reference lists of all included articles were manually reviewed to identify additional publications addressing the welcoming of migrant children in PHC.

### Data Extraction and Analysis

The study selection process was conducted independently and in a blinded manner by two reviewers, with a third reviewer consulted in cases of disagreement. The reasons for excluding full-text sources of evidence that did not meet the inclusion criteria were recorded and reported in the scoping review. Subsequently, the selected articles were analyzed in full before being included in the final sample.

For data extraction, information on the author, year, country, study type, level of evidence established by the JBI^(11)^, participants, and welcoming care practices directed toward migrant children in PHC was considered. This evidence was descriptively organized in a summary chart (Chart 2), developed by the authors and adapted according to the recommendations of the JBI manual^(8)^.

**Chart 2:** Characteristics of the studies included in the final review sample – Niterói, RJ, Brazil, 2026.

Code	Author Year Country	Study design Level of evidence	Participants	Care practices
*A1^(51)^	Nascimento et al. 2024 Brazil	Clinical trial study 2.b	Resident and transient populations in Brazilian border areas, including children, Brazilians, and foreigners (number of participants not specified)	Vaccination
A2^(13)^	Ouzounidou et al. 2024 Greece	Quantitative and descriptive cross-sectional study 4.b	54 physicians, 43 nurses, and 23 community health agents	Vaccination Appointments
*A3^(14)^	Ayoub-Rodriguez et al. 2024 USA	Opinion article 5.b	Migrant children (number of participants not specified)	Appointments Medication administration
A4^(15)^	Reda et al. 2023 Norway	Clinical trial study 5.b	300 participants (approximately 50 parent-child pairs from each center)	Dental follow-up Anthropometric assessment
A5^(16)^	Yalçın et al. 2023 Turkey	Descriptive study 4.b	876 Syrian physicians/nurses	Appointments Health guidelines
A6^(17)^	Kvamme and Voldner 2022 Norway	Descriptive and qualitative study 6.b	Seven public health nurses	Appointments
A7^(18)^	Shon et al. 2020 South Korea	Methodological study with usability testing 4.b	Eight community nurses and 261 children	Appointments Anthropometric assessment Laboratory tests
A8^(19)^	Salami et al. 2020 Canada	Qualitative and descriptive study 6.b	17 fathers and 33 mothers	Appointments Bond
A9^(20)^	Zanatta et al. 2020 Brazil	Exploratory, descriptive study with a qualitative approach 6.b	Ten nurses	Appointments Home visits Anthropometric assessment Health guidelines Nutritional follow-up
A10^(21)^	Ragavan et al. 2020 USA	Quantitative, cross-sectional study 4.b	212 parents	Appointments
A11^(22)^	Shin et al. 2019 South Korea	Descriptive and exploratory study using a mixed-methods approach that included a literature review 4.b	16 local residents, 18 public healthcare professionals (nurses and physicians), eight community leaders, and 141 adults from each family	Appointments Home visit
A12^(23)^	Shin et al. 2019 Kyrgyzstan	Retrospective evaluative study of a pilot project 3.b	85 children	Anthropometric assessment Home visit Laboratory tests Dietary supplementation Appointment Screening Health guidelines
A13^(24)^	Oldfield et al. 2019 USA	Descriptive qualitative study with content analysis 6.b	22 caregivers	Appointment Health guidelines
A14^(25)^	Schrier et al. 2019 Europe	Systematic and comparative review study 5.b	Migrant and refugee children (number of participants not specified)	Health guidelines Anthropometric assessment Appointments Vaccination Laboratory tests
A15^(26)^	Pascual et al. 2018 Spain	Qualitative and descriptive study 6.b	17 healthcare professionals and seven parents	Appointments Health guidelines
A16^(27)^	Bull et al. 2018 USA	Quality improvement project study 3.b	68 patients registered at the project site	Anthropometric assessment Nutritional follow-up Growth and development follow-up Appointments
A17^(28)^	Carrasco-Sanz et al. 2018 Europe	Quantitative cross-sectional study 4.b	492 pediatricians	Vaccination Appointments
A18^(29)^	Sami et al. 2018 South Sudan	Case study 4.b	104 healthcare professionals and seven senior managers	Appointments Referral to specialized services
A19^(30)^	Caballero et al. 2017 USA	Narrative review and critical synthesis study 5.b	Migrant children and adolescents	Psychological follow-up Appointments
A20^(31)^	Arcury et al. 2016 USA	Quantitative, observational, and cross-sectional study 4.b	221 mothers of 221 children	Appointments Anthropometric assessment Nutritional follow-up
A21^(32)^	Fagerlund et al. 2016 Norway	Qualitative and descriptive study 6.b	26 public health nurses	Nutritional follow-up Appointments Health guidelines Anthropometric assessment
A22^(33)^	Cyril et al. 2016 Australia	Qualitative and exploratory study 6.b	59 service providers involved in child health and well-being	Nutritional follow-up Anthropometric assessment Appointments
*A23^(34)^	Seery et al. 2015 USA	Literature review study 5.b	Refugee children (number of participants not specified)	Health assessment Appointments Laboratory tests Nutritional follow-up Anthropometric assessment Vaccination Psychological follow-up
A24^(35)^	McMurray et al. 2014 Canada	Before-and-after study 3.b	872 children/adults	Appointments Referral to specialized services
*A25^(36)^	Measham et al. 2014 Canada	Literature review study 5.b	Refugee children and refugee families (number of participants not specified)	Psychological follow-up Referral to specialized services Appointments
*A26^(37)^	Huang and Au 2014 USA	Experience report study 5.b	Chinese migrant families and children with special health needs (number of participants not specified)	Appointments Referral to specialized services
*A27^(38)^	Rousseau et al. 2013 Canada	Exploratory qualitative study 5.b	Refugee children (number of participants not specified)	Social follow-up Psychological follow-up Appointments
*A28^(39)^	Chilton et al. 2013 USA	Literature review study 5.b	Migrant children and residents of border regions (number of participants not specified)	Appointments Anthropometric assessment Referral to specialized services
*A29^(40)^	Capella 2012 Spain	Theoretical-conceptual study with a qualitative approach, of the bioethical essay/reflection type 5.b	Children of international origin (number of participants not specified)	Appointments Bond
*A30^(41)^	Woodland et al. 2010 Australia	Development of a model based on national and international literature and current service models 5.b	Newly arrived refugee children and their families (number of participants not specified)	Health screening Appointments
*A31^(42)^	Vostanis 2010 United Kingdom	Qualitative, theoretical-reflective study involving review and conceptual discussion 5.b	Children and adolescents belonging to vulnerable groups (number of participants not specified)	Appointments Psychological follow-up
A32^(43)^	Samarasinghe et al. 2010 Sweden	Qualitative, descriptive, and exploratory study 6.b	34 public health nurses	Appointments Health guidelines Nutritional follow-up Home visits
A33^(44)^	Swe and Ross 2010 USA	Exploratory study 6.b	36 children/adolescents/adults/older adult	Health screening Vaccination Appointments Use of various healthcare services
A34^(45)^	Swingle et al. 2008 USA	Observational mixed-methods (quantitative-qualitative) study 4.b	71 families	Appointments
*A35^(46)^	Paris and Bronson 2006 USA	Descriptive study (experience report/intervention program description) with a qualitative approach 5.b	Migrant and refugee women considered high-risk	Home visits
A36^(47)^	Melvin 2006 USA	Descriptive study (experience report/program assessment), observational and longitudinal, using a before-and-after comparison 5.b	500 children	Dental services Appointments
*A37^(52)^	Grant et al. 2005 Australia	Literature review 5.b	Children, families, and healthcare professionals	Health guidelines
A38^(53)^	Ekezie et al. 2002 Germany	Systematic review study 4.b	104 studies analyzed	Vaccination Health guidelines
A39^(48)^	Clark 2002 USA	Qualitative study (focused ethnography) 6.b	25 mothers	Appointments Vaccination
*A40^(49)^	Wilson et al. 2000 USA	Descriptive study (experience report) with a quantitative and qualitative approach 6.b	Hispanic children of migrant farmworkers	Nursing interventions Appointments Health guidelines Anthropometric assessment Laboratory tests Referral to specialized services
A41^(54)^	Wilson et al. 2000 USA	Qualitative, descriptive, exploratory study 5.b	73 children	Laboratory tests Health guidelines
A42^(50)^	Doering et al. 1999 Canada	Descriptive study of the experience report type 4.b	1,230 children	Appointments Laboratory tests

EUA – United States of America;

*The articles do not describe the number of participants.

An inductive thematic analysis was conducted, with categories emerging from the data. The categorization process was guided by Bardin’s theoretical-analytical framework^(12)^. The analytical categories were not predefined but emerged from the reading and interpretation of the data extracted from the included studies.

The analytical process was conducted in three stages: pre-analysis, consisting of an exhaustive reading of the selected articles; exploration of the material through the identification and coding of recurring themes related to welcoming practices for migrant children in PHC; and data treatment and interpretation, involving the grouping of codes according to thematic similarity and the development of the analytical categories.

The analysis was conducted independently by two reviewers, with a third reviewer consulted in cases of disagreement. Disagreements were resolved by consensus in order to ensure methodological rigor, analytical traceability, and the reduction of interpretive bias.

This process resulted in the development of three emerging thematic categories: “Clinical and Follow-up Actions in Primary Health Care”; “Preventive Health Actions in Primary Health Care”; and “Health Care Network Coordination”.

## RESULTS

### General Characteristics of Studies

**Figure 1:**
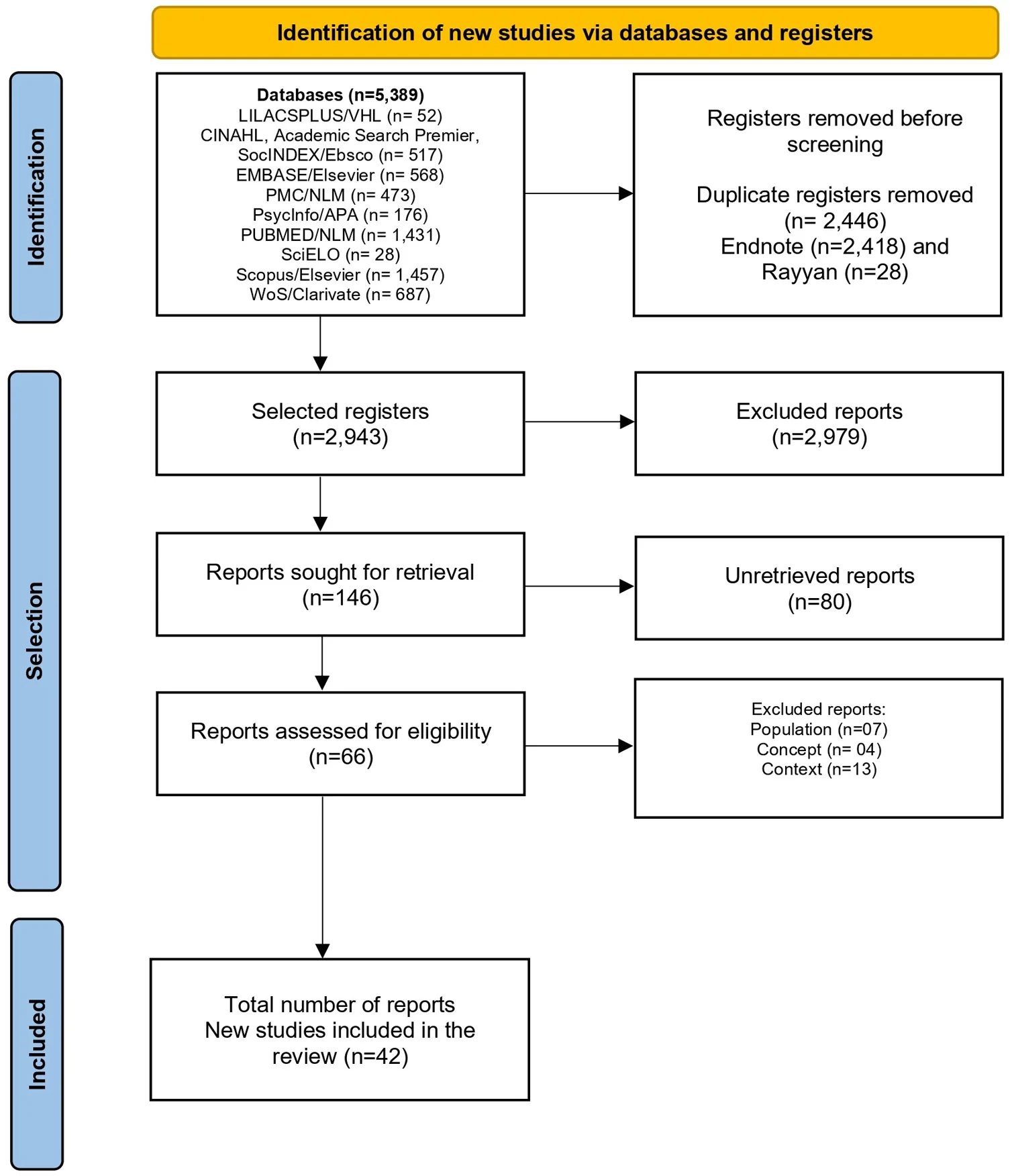
Preferred Reporting Items for Systematic Reviews and Meta-Analyses extension for scoping reviews flow diagram of the publication selection process for the review – Niterói, RJ, Brazil, 2026.

The database search was conducted in May 2024, when 5,389 records were identified from searches performed in the Scopus (n = 1,457), PubMed (n = 1,431), Web of Science (n = 687), EMBASE (n = 568), EBSCO – CINAHL, Academic Search Premier, and SocINDEX (n = 517), PMC (n = 473), PsycINFO (n = 176), BVS (n = 52), and SciELO (n = 28) databases. After duplicate records were removed, studies were screened based on titles, abstracts, and full-text review. Although a manual search of the reference lists was performed, no additional eligible studies were identified. Based on the inclusion criteria, 42 records were selected for the scoping review (Figure 1).

The analyzed articles were published between 1999 and 2024, demonstrating a broad period of scientific production on the topic. The most frequent years of publication were 2010, 2018, 2019, and 2020. Regarding the distribution of studies by country, there was a marked concentration of scientific production in the United States, with 16 articles. Canada ranked second, with five articles. Qualitative research predominated, representing the most frequent study design included in this scoping review. Overall, 15 studies adopted a qualitative approach, including descriptive, exploratory, interpretative, ethnographic, and content analysis designs.

Regarding the level of evidence of the 42 included studies, studies with a low potential for causal inference predominated, particularly qualitative and descriptive research, demonstrating that the scientific production on the welcoming of migrant children in PHC remains predominantly exploratory and focused on understanding experiences and care practices. For the question of meaningfulness, most of the evidence was classified as level 3, consisting of single qualitative studies exploring welcoming experiences. For the question of effectiveness, the studies were predominantly classified as level 4, represented by descriptive observational and cross-sectional designs. The concentration of evidence at these levels reflects the nature of the study object, which focuses on complex social and care contexts in which controlled clinical trials are less common.

**Figure 2:**
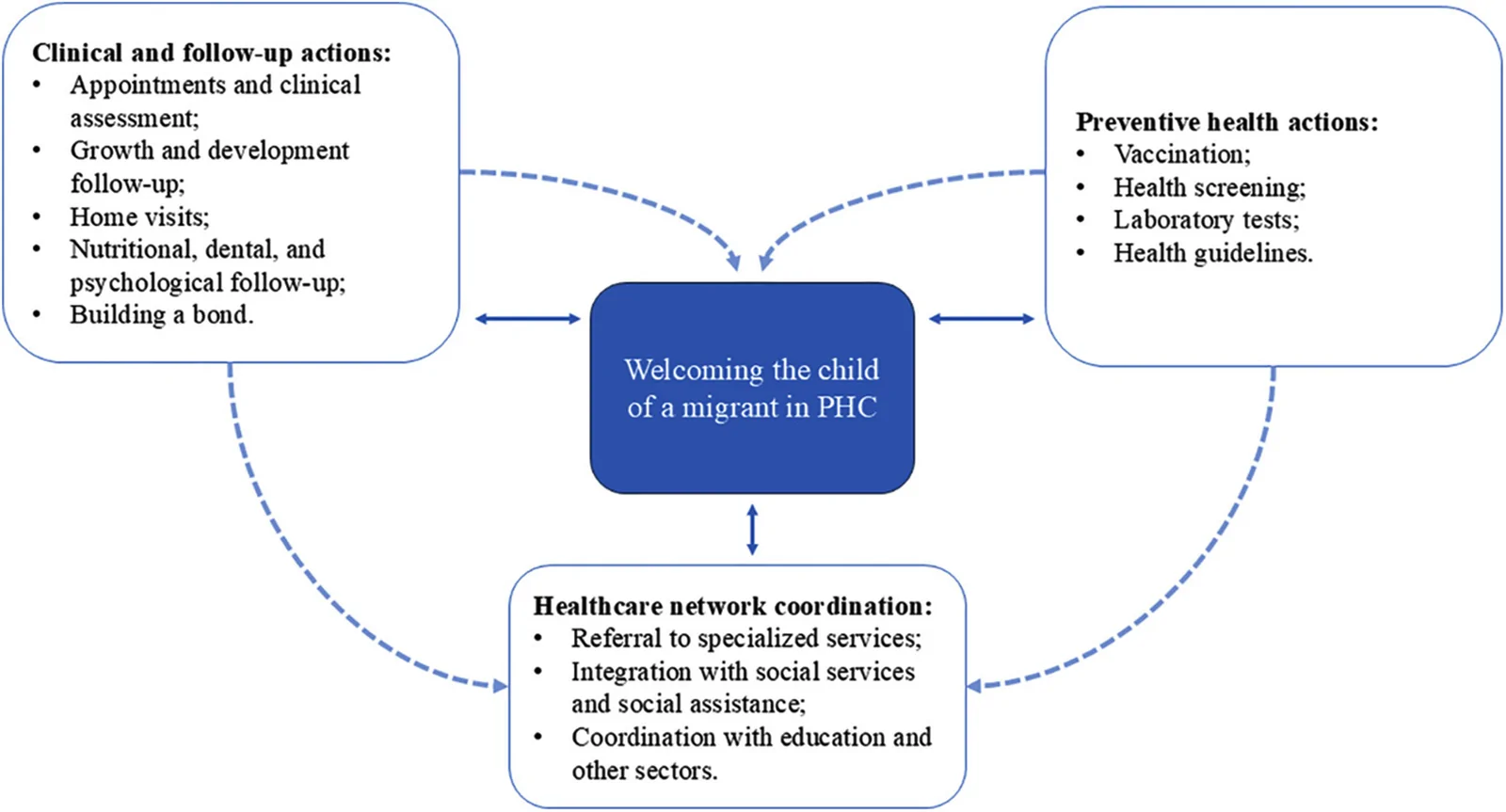
Conceptual diagram of reception and care practices for migrant children in Primary Health Care.

The welcoming care practices directed toward migrant children were organized into three analytical categories. To synthesize these findings, Figure 2 presents a conceptual model illustrating the central categories, their dimensions, and the interrelationships identified in this scoping review.

### Clinical and Follow-up Actions in Primary Health Care

Thirty-eight articles addressing clinical and follow-up actions carried out in PHC were identified^(13,14,15,16,17,18,19,20,21,22,23,24,25,26,27,28,29,30,31,32,33,34,35,36,37,38,39,40,41,42,43,44,45,46,47,48,49,50)^. These actions included appointments with higher education healthcare professionals^(13,14,15,16,17,18,19,20,21,22,23,24,25,26,27,28,29,30,31,32,33,34,35,36,37,38,39,40,41,42,43,44,45,47,48,49,50)^, focusing on clinical assessment, growth and development monitoring, management of chronic and acute conditions, and nutritional, dental, and psychological follow-up.

Home visits^(20,22,23,43,46)^ were also described as practices that contribute to understanding the living conditions of migrant families and strengthening the bond^(19,40)^ between healthcare professionals and users.

### Preventive Health Actions in Primary Health Care

This category included 21 articles^(13,16,18,20,23,24,25,26,28,32,34,41,43,44,48,49,50,51,52,53,54)^ reporting preventive health actions in PHC, with vaccination^(13,25,28,34,44,48,51,53)^ and health screening^(23,41)^ standing out, particularly among newly arrived migrant children. During this initial period in the host country, migrant children also undergo laboratory tests^(18,23,25,34,49,50,54)^ to screen for infectious diseases.

Moreover, health education^(16,20,23,24,25,32,43,49,52,53,54)^ was described as a cross-cutting welcoming strategy aimed at health promotion, disease prevention, and supporting newly arrived migrant families in their use of healthcare services.

### Healthcare Network Coordination

This final category comprised six articles addressing healthcare network coordination^(29,35,36,37,39,49)^, which was identified as an essential component of comprehensive care for migrant children. The studies reported referrals to specialized, social, and educational services, highlighting the need for comprehensive and intersectoral care to address the multiple needs of these migrant children.

## DISCUSSION

In general, the welcoming care practices provided by healthcare professionals to migrant children in PHC were evidenced through follow-up actions, preventive actions, and coordinated referrals within the healthcare network. Therefore, user embracement is not characterized solely as an organization of the care flow but also as a technology used to ensure access to therapeutic care within PHC^(55)^.

Clinical and follow-up actions were the practices most frequently identified, including multiprofessional appointments, clinical assessment, growth and development monitoring, anthropometric assessment, nutritional and psychological follow-up, and home visits. These findings confirm that PHC constitutes the main entry point for child healthcare in different healthcare systems, although this access may be limited for migrant populations in certain contexts. In this regard, the operationalization of these services requires the systematic mapping of territorial challenges and facilitating factors that promote equity in access^(56,57)^.

The predominance of appointments and growth and development monitoring reflects care centered on the biomedical and programmatic model of healthcare for migrant children, prioritizing the early diagnosis of health problems and longitudinal follow-up. However, with regard to migrant populations, studies indicate that these populations face linguistic, cultural, and socioeconomic barriers that may compromise the effectiveness of these actions, highlighting the need for culturally sensitive and family-centered approaches^(58)^.

Home visits emerged as a relevant strategy for strengthening bonds and understanding the family and social context and are recognized as an essential tool in PHC for identifying the social determinants of health. At the same time, the literature indicates that this practice is fundamental to understanding the migrant population and registering them within the territory; as professionals identify this group as either a permanent or transient part of the area, it becomes necessary to adopt strategies that promote their reception and integration into the unit’s care workflow^(59)^.

The importance of PHC and its central role in child health should be emphasized, as it represents the main entry point to the SUS. PHC encompasses health actions at the individual, family, and community levels, including health promotion, disease prevention, health protection, diagnosis, treatment, rehabilitation, harm reduction, palliative care, and health surveillance, all of which should be delivered through comprehensive care and qualified management^(56)^.

Preventive actions carried out by healthcare professionals, especially vaccination, health screening, laboratory testing, and health education, were widely described in the included studies. Vaccination was highlighted as one of the main welcoming strategies. Studies indicate that immunization is the primary strategy for the prevention and control of vaccine-preventable diseases, as it constitutes an essential public health action^(60)^.

In Brazil, vaccination is constitutionally guaranteed, including for newborns of migrant parents, as it is considered a national health indicator. In addition, border regions are supported by vaccination services that provide immunization. During this initial contact, migrant populations entering the country undergo health screening and vaccination^(61)^.

In the Brazilian and Latin American contexts, the applicability of these findings should be interpreted with caution, considering the specific characteristics of healthcare systems, social inequalities, and regional migration flows. Although the SUS advocates universal access, structural, organizational, and sociocultural barriers may still hinder access to and continuity of care for migrant populations. Therefore, strategies described in the international literature may not be directly transferable, requiring adaptations that take into account structural inequalities, SUS organization, and the specific characteristics of regional migration flows.

Health education and educational activities were described as cross-cutting strategies aimed at health promotion and supporting migrant children in their use of healthcare services. These activities should primarily consider the cultural and linguistic characteristics, as well as the sociocultural trajectory, of this population, without prejudice or stigma that may interfere with the provision of care^(59)^.

Interactions between healthcare professionals and children should occur positively for both parties; however, studies have reported recurrent negative experiences. Critical reflections that consider the individual, cultural, and socioeconomic needs of migrants are also rare. Therefore, the relationship between healthcare professionals and migrants tends, in some cases, to be conflictive and with limited resolution of the reported health needs and problems^(62)^.

Therefore, to ensure that the reported needs are addressed, it is necessary to rethink work processes based on the recognition and situational diagnosis of the territory, outlining strategies and considering the difficulties that restrict migrant children’s access to healthcare services. In addition, it is essential to ensure and improve healthcare provided to migrant users at all levels of healthcare for this population^(59)^.

Healthcare network coordination was the least frequent category among the included studies and was reported mainly through referrals to specialized, social, and educational services. This finding demonstrates that, although recognized as essential for comprehensive care, intersectoral integration is still poorly described and operationalized in the literature on migrant children in PHC. Studies indicate that intersectoral support, when tailored to the needs of migrant families, can improve the health promotion of migrant children^(62)^.

Against this background, intersectorality can be understood as a fundamental strategy for overcoming the fragmentation of knowledge and social policies through the articulation among different sectors. This integration enables the development of joint and complementary actions with the potential to address health inequities and improve the living conditions of populations^(63)^.

The relationship between health and migration presents a complex dynamic for those who experience this entire process. Although this is not universally the case, part of the migrant population may be exposed to health problems and precarious living conditions^(59)^, making it necessary for PHC professionals to be willing to provide user embracement.

Although the findings of this review highlight the central role of PHC in the care of migrant children, it is important to consider the limitations of the included studies. Qualitative, descriptive, and observational studies predominated and were mostly classified as providing moderate and low levels of evidence according to the JBI criteria. This finding demonstrates that the scientific literature on the welcoming of migrant children in PHC remains focused on understanding experiences, perceptions, and care practices, with a scarcity of analytical or interventional studies capable of producing evidence with greater potential for generalization and causal inference.

Furthermore, the possible presence of publication bias should be considered, as studies reporting successful user embracement experiences tend to be published more frequently than studies revealing weaknesses or shortcomings in healthcare.

The complexity of the migration process and its impacts on child health reinforce the need to strengthen welcoming practices in PHC, incorporating approaches that consider social, cultural, and structural determinants. However, important gaps remain in the literature regarding the effectiveness of these strategies, especially in low- and middle-income countries. In this regard, future studies with greater methodological rigor, as well as public policies that promote equity in access to and the quality of care provided to migrant children, are needed.

Although the international literature presents established models of clinics dedicated to refugees in the Northern Hemisphere, the applicability of these findings to the SUS and other Latin American contexts requires careful analysis. Unlike insurance-based systems or isolated reception centers, user embracement in Brazil should be operationalized within the universal PHC network, while avoiding fragmentation of care. Thus, welcoming practices in border territories, such as Roraima, should focus on strengthening Basic Health Units as the usual source of care, integrating epidemiological surveillance with the cultural sensitivity required to provide care for Venezuelan families.

## CONCLUSION

This scoping review mapped the welcoming care practices directed toward migrant children in PHC, demonstrating that user embracement has been operationalized predominantly through clinical and follow-up actions, preventive actions, and healthcare network coordination.

The findings reinforce the central role of PHC as the preferred entry point for child healthcare and as the coordinator of the healthcare network, highlighting user embracement as a fundamental relational and organizational technology to ensure access to and continuity of care for migrant children. Intersectoral coordination with social assistance, education, and social protection services was the least frequently described category among the included studies, indicating weaknesses in care coordination and in the implementation of integrated public policies.

Therefore, there is a clear need for future studies addressing culturally competent welcoming strategies, structured intersectoral interventions, and the assessment of the effectiveness of care practices directed toward migrant children in PHC. Accordingly, future research is recommended to deepen the understanding of this population’s needs and to support the development of more integrated, equitable, and child- and family-centered policies and care practices.

## Data Availability

The complete dataset supporting the findings of this study is available within the article itself.
