# Supplementary material for: Welcoming care practices for migrant children in Primary Health Care: a scoping review
Source: Rev Esc Enferm USP. 2026 Aug 31;60:e20260121. doi: 10.1590/1980-220X-REEUSP-2026-0121en (PMC13553000; doi:10.1590/1980-220X-REEUSP-2026-0121en)
Supplement: Translation

# Translation: Práticas assistenciais de acolhimento para crianças migrantes na Atenção Primária à Saúde: revisão de escopo

**DOI:** 10.1590/1980-220X-REEUSP-2026-0121pt

**Authors:** Ana Beatriz Oliveira Costa, Lanna Jeniffer Silva Rodrigues, Mônica Letícia Martins Franco, Paulo Sérgio da Silva, Audrey Vidal Pereira, Liliane Faria da Silva

**Affiliations:** 1 Universidade Federal Fluminense, Programa Acadêmico em Ciências do Cuidado em Saúde, Niterói, RJ, Brasil.; 2 Universidade Federal de Lavras, Faculdade de Ciências da Saúde, Lavras, MG, Brasil.

**Keywords:** Acolhimento, Atenção Primária à Saúde, Assistência Integral à Saúde, Migrantes

## Abstract

**Objetivo:**

Mapear e sintetizar as evidências disponíveis sobre as práticas assistenciais de acolhimento direcionadas às crianças migrantes na Atenção Primária à Saúde.

**Método:**

Revisão de escopo conduzida segundo as recomendações do JBI e descrita de acordo com o checklist PRISMA-ScR. As buscas foram realizadas nas bases MEDLINE/PubMed, CINAHL, *Academic Search Premier*, *Academic Search Ultimate* (EBSCO), Scopus, EMBASE/Elsevier, APA *PsycInfo* (BVS), LILACS, BDENF, *Index Psychology – Periodicals* e SciELO.

**Resultados:**

Foram incluídos 42 estudos publicados entre 1999 e 2024. As evidências foram categorizadas em ações clínicas e de acompanhamento na Atenção Primária à Saúde, ações preventivas em saúde na Atenção Primária à Saúde e articulação da rede de saúde. Os estudos incluídos apresentaram heterogeneidade quanto aos delineamentos metodológicos, contextos de realização e características das populações investigadas.

**Conclusão:**

O acolhimento tem se concentrado em ações clínicas, preventivas e de articulação na rede, reafirmando a Atenção Primária à Saúde como principal porta de entrada do cuidado infantil. Contudo, revelam a necessidade de estudos futuros que abordem estratégias de acolhimento culturalmente competentes, intervenções intersetoriais estruturadas e a avaliação da efetividade dessas práticas.

## INTRODUÇÃO

Inicialmente, é oportuno contextualizar que o fenômeno migratório consiste no deslocamento populacional entre diferentes regiões. Quanto à sua abrangência espacial, a migração pode ser classificada como interna, quando restrita aos limites territoriais de um estado soberano, ou internacional, quando envolve a transposição de fronteiras nacionais e o estabelecimento em um país estrangeiro^(1)^. De fato, o evento migratório influencia diretamente a saúde do migrante, e o processo de adaptação ao novo local nem sempre é positivo. A pessoa migrante pode se tornar vulnerável devido à sua condição social e, em alguns casos, enfrentar isolamento e afastamento familiar, o que aumenta a probabilidade de sofrer estigma, discriminação e preconceito^(2)^.

As pessoas migrantes, ao adentrarem um novo país, deparam-se com barreiras no acesso aos serviços de saúde, incluindo a ausência de seguro de saúde, o receio de deportação, a exclusão de programas governamentais e as diferenças linguísticas e culturais^(3)^. Especificamente, as crianças em situação de mobilidade apresentam uma elevada vulnerabilidade social e estão expostas a riscos severos, como insegurança alimentar, tráfico humano, exploração sexual e negligência nos cuidados a saúde. Nesse sentido, o processo migratório, que compreende o deslocamento e a transposição de fronteiras sob repressão, além da complexa readaptação sociocultural, submete a criança a eventos estressores com repercussões profundas e duradouras sobre sua saúde mental^(4)^.

Diante dessas pontuações e considerando as especificidades da população infantil migrante, a Atenção Primária à Saúde (APS) é uma estratégia central no campo da saúde pública, sobretudo por monitorar em caráter longitudinal o crescimento e o desenvolvimento das crianças, estimular a participação comunitária nos serviços de saúde e contribuir para a promoção do bem-estar das populações. Além disso, é indispensável situar que a APS contribuiu para o fortalecimento das relações entre a comunidade e os atores do sistema de saúde, o que favorece o empoderamento em âmbito local e regional do Sistema Único de Saúde (SUS)^(5)^.

Nessa lógica, as necessidades em saúde da população infantil migrante no âmbito da APS podem ser reconhecidas por meio do acolhimento em saúde, compreendido como um dispositivo que reconhece o usuário e suas necessidades de saúde como legítimas e singulares, sustentando a dimensão relacional e humana do processo de cuidado. O ato de acolher pode envolver outras ferramentas do cuidado, como a escuta qualificada, que contribui para a construção de vínculos e para uma compreensão mais aprofundada das necessidades migratórias^(6)^.

No Brasil, ao longo dos anos, a saúde infantil tem apresentado avanços na organização do cuidado no âmbito da APS. Essa melhoria na qualidade da assistência é resultado da consolidação dos direitos legais das crianças e da evolução das políticas públicas de saúde no contexto nacional. A criança na APS recebe ações assistenciais fundamentadas nos princípios do direito universal à vida, da equidade, da integralidade do cuidado, da humanização, da atenção e da gestão participativa^(7)^.

Com base nessas preliminares considerações, foi realizada, em abril de 2024, uma busca preliminar no *International Prospective Register of Systematic Reviews* (PROSPERO) e nas principais bases de dados, não sendo identificada qualquer revisão de escopo ou sistemática, publicada ou em andamento, relacionada às especificidades da população infantil migrante. No contexto apresentado, pouco se fala de maneira detalhada acerca das práticas assistenciais de acolhimento para crianças migrantes prestadas pelos profissionais de saúde no âmbito da APS.

Nessa perspectiva, foi realizada uma revisão de escopo (em inglês, *scoping review*) com os objetivos de mapear e sintetizar as evidências disponíveis sobre as práticas assistenciais de acolhimento direcionadas às crianças migrantes na APS. A partir deles, buscou-se identificar lacunas, promover a equidade no acesso à saúde, apoiar políticas públicas e melhorar a qualidade do atendimento a crianças migrantes na atenção primária, de modo a garantir um cuidado culturalmente sensível às necessidades da população infantil migrante.

## MÉTODO

### Desenho do Estudo

Trata-se de uma revisão de escopo, elaborada de acordo com o referencial teórico disponibilizado pelo JBI^(8)^. Este tipo de revisão objetiva mapear as principais evidências científicas e as limitações de um determinado tema disponível na literatura, com as recomendações propostas pelo *Preferred Reporting Items for Systematic reviews and Meta-Analyses extension for Scoping Reviews* (PRISMA-ScR), a partir de uma extensão para revisão de escopo^(9)^. Durante a fase exploratória da revisão, teve-se como objetivo verificar a inexistência de relatórios de pesquisa ou protocolos de revisão recentes que abordassem uma temática semelhante à do presente estudo. Posteriormente, o protocolo desta revisão foi registrado na plataforma *Open Science Framework* (https://doi.org/10.17605/OSF.IO/ZSTNX).

Considerando que se trata de uma revisão de escopo, cujo objetivo é mapear e caracterizar as evidências disponíveis na literatura, não foi realizada avaliação crítica formal da qualidade metodológica dos estudos incluídos. Essa decisão está alinhada às recomendações do JBI, que não torna obrigatória a avaliação da qualidade em revisões de escopo, especialmente quando os focos são a amplitude da evidência e a identificação de lacunas de conhecimento.

### Identificação da Questão de Pesquisa

Para a elaboração da questão de pesquisa, adotou-se o método População, Conceito e Contexto (PCC), uma estratégia que auxilia na identificação dos tópicos-chave e é recomendada para a condução de revisões de escopo. Logo, o desenvolvimento deste estudo ocorreu a partir da seguinte questão: quais são as práticas de acolhimento para crianças migrantes de 0 a 10 anos na APS?

Foram incluídos estudos que tenham crianças de 0 a 10 anos como população-alvo, com enfoque específico para migrantes. Neste estudo, são considerados migrantes as pessoas que se deslocam de seu local de residência habitual, seja dentro de um país ou através de uma fronteira internacional, temporária ou permanentemente^(10)^.

### Critérios de Seleção

Esta revisão de escopo abrangeu estudos experimentais e quase-experimentais, incluindo ensaios clínicos randomizados, ensaios clínicos não randomizados, estudos antes e depois e estudos de séries temporais interrompidas. Além disso, foram incluídos estudos observacionais analíticos do tipo coorte prospectivos e retrospectivos, estudos de caso-controle e estudos transversais. Também foram incluídos estudos observacionais descritivos, como séries de casos, relatos de casos individuais e estudos transversais descritivos. Estudos qualitativos também foram incluídos, abrangendo diferentes abordagens, como fenomenologia, teoria fundamentada, etnografia, descrição qualitativa, pesquisa-ação e pesquisa feminista. Revisões de qualquer tipo que atendam aos critérios de inclusão foram consideradas em convergência com a questão de pesquisa. Por fim, textos e artigos de opinião também foram incluídos nesta revisão de escopo. Foram excluídos resumos e cartas ao editor.

Considerando que se trata de uma revisão de escopo, cujo objetivo é mapear e caracterizar as evidências disponíveis na literatura, não foi realizada avaliação crítica formal da qualidade metodológica dos estudos incluídos. Essa decisão está alinhada às recomendações do JBI, que não torna obrigatória a avaliação da qualidade em revisões de escopo, especialmente quando o foco é a amplitude da evidência e a identificação de lacunas de conhecimento.

### Estratégia de Busca

A estratégia de busca foi conduzida com o objetivo de localizar estudos publicados e não publicados. Inicialmente, a busca preliminar foi realizada considerando os termos dos Descritores em Ciências da Saúde, dos *Medical Subject Headings* e dos *Embase Subject Headings*, que foram combinados pelos operadores booleanos *OR* e *AND* no MEDLINE/PubMed, OSF, PROSPERO, *JBI Synthesis* e *Cochrane Library*. O objetivo era identificar protocolos, revisões e artigos primários, novas palavras-chave e termos de indexação padronizados para uma estratégia abrangente.

Secundariamente, os termos identificados compuseram a estratégia de busca definitiva, que foi adaptada às bases de dados e às fontes de informação definidas para a revisão, a saber: MEDLINE/PubMed; *Cumulative Index to Nursing and Allied Health Literature* (CINAHL); *Academic Search Premier* e *Academic Source* da EBSCO; Scopus; EMBASE da Elsevier; e APA *PsycInfo*. Também foram pesquisadas as bases de dados do Portal Regional da Biblioteca Virtual em Saúde (BVS): Literatura Latino-Americana e do Caribe em Ciências da Saúde (LILACS), Base de Dados de Enfermagem (BDENF) e *Index Psychology – Periodicals*, bem como o portal de periódicos *Scientific Electronic Library Online* (SciELO). Foram incluídos estudos sem restrições de idioma e sem limite temporal.

O Quadro 1 apresentou as estratégias de busca adotadas em cada base de dados, organizadas conforme o acrômio PCC, com descritores e palavras-chave dispostos por bloco, explicitação da lógica booleana e número de registros encontrados.

**Quadro 1:** Estratégia de busca conduzida em 10 de maio de 2024 para cada base de dados – Niterói, RJ, Brasil, 2024.

Bases de dados	Estratégias de busca	Registros encontrados (n)
LILACSPLUS/BVS	Migrantes OR Migrante OR Nômade OR Nômades OR Nomadismo OR Não-migrante OR Não-migrantes OR Posseiro OR Posseiros OR Transeunte OR Transeuntes OR Nómada OR Nómadas OR Nomadismo OR Ocupante OR Migratorio OR Population de Passage et Migrants OR Migrants OR Nomade OR Population de passage OR Population en Transit OR Population en Transit et Migrants OR Sédentaires OR Squatters OR Squatteurs OR Squatteuses OR Transients and Migrants OR Migrant OR Nomad OR Nonmigrant OR Squatter OR Transien; AND Criança OR Infantil OR Infancia OR Infant, Newborn OR Newborn Infant OR Newborn OR Neonate OR Infant OR Child, Preschool OR Preschool OR Full Term Infant OR Neonatus OR Newly Born Baby OR Newly Born Child OR Newly Born Infant OR Pediatric OR Infant, Premature OR Preterm OR Premature OR Neonatal Prematurity OR Prematurita OR Recem-Nascido OR Neonato OR Lactente OR Prematuro OR Infancia OR Cuidado do Lactente OR Cuidado del Lactante OR Atención del Lactante OR Soins du Nourrisson OR Soins au Nouveau-né et au Nourrisson OR Soins aux Nouveau-nés et aux Nourrissons OR Soins des Nourrissons OR Soins Infantiles OR Cuidado da Criança OR Cuidado Infantil OR puericultura OR Cuidado del Niño OR Cuidado Infantil OR Puericultura OR Soins de L’enfant OR Soins aux Enfants OR Soins des Enfants OR Infant Care OR Child Care OR Child Day Care OR puericulture; AND Acolhimento OR User Embracement OR Acogimiento OR Adoption par l’Utilisateur OR Amparo OR Assistência OR Abrigamento OR Cuidado OR Support OR Care OR Welcoming OR Apoyo OR Asistencia OR Bienvenida OR Integración OR Adoption par l’Utilisateur OR Accueil OR Soutien OR Assistance OR Bienvenue OR Intégration; OR Criança Acolhida OR Criança Bem-Vinda OR Criança em Regime de Acolhimento OR Crianças Acolhidas OR Crianças Bem-Vindas OR Crianças em Regime de Acolhimento OR Jovem Acolhido OR Jovem Bem-Vindo OR Jovens Acolhidos OR Jovens Bem-Vindos OR Niño Acogido OR Joven Acogido OR Jóvenes en Régimen de Acogida OR Niña Acogida OR Niños Acogidos OR Enfant Placé en Famille d’accueil OR Enfant Accueilli OR Enfants accueillis OR Enfants Placés en Familles D’accueil OR Jeune Accueilli OR Jeune Placé en Famille D’accueil OR Child, Foster OR Foster Child OR Foster Children OR Foster Youth OR Foster Youths; AND Atenção Primária à Saúde OR Atención Primaria de Salud OR Atendimento Básico OR Atendimento Primário OR Atenção Básica OR Atenção Básica de Saúde OR Atenção Básica à Saúde OR Atenção Primária OR Atenção Primária de Saúde OR Atenção Primária em Saúde OR Cuidados Primários OR Cuidados Primários de Saúde OR Cuidados Primários à Saúde OR Cuidados de Saúde Primários OR Centros de Saúde OR Centros de Salud OR Centro de Saúde OR Posto de Assistência Médica OR Posto de Saúde OR Postos de Saúde OR Unidade Básica de Saúde OR Serviços Básicos de Saúde OR Servicios Básicos de Salud OR Prestações Básicas de Saúde OR Unidade de Saúde OR Unidade de Serviço OR Prestaciones Básicas de Salud OR Centro de Salud OR Postas Médicas OR Puestos Médicos OR Puestos de Salud OR Primary Health Care OR Primary Healthcare OR Primary Care OR Health Centers OR Health Center OR Health Posts OR Basic Health Services OR Basic Health Care Provisions OR Community Health Services OR Community Health Care OR Community Health Service OR Community Healthcare OR Assistência à Saúde Comunitária OR Assistência Médica Comunitária OR Cuidados Médicos Comunitários OR Atención de la Salud Comunitaria OR Atención Médica Comunitaria OR Services de Santé Communautaires OR Services de Santé Communautaire OR Services de Santé de Proximité OR Soins de Santé Communautaires OR Soins de Santé de Proximité; AND Instance: lilacsplus.	52
CINAHL e SocINDEX/EBSCO	Puericulture OR Childcare OR Infant, Newborn OR Newborn Infant OR Newborn OR Neonate OR Infant OR Infant OR Child, Preschool OR Preschool OR Full Term Infant OR Neonatus OR Newly Born Baby OR Newly Born Child OR Newly Born Infant OR Pediatric OR Infant, Premature OR Preterm OR Premature OR Neonatal Prematurity OR prematurita OR Child OR Child OR Children; AND User Embracement OR support OR care OR welcoming OR Embracement; OR Child, Foster OR Foster Child OR Foster Youth OR Infant Care OR Child Care OR Child Day Care; OR Puericulture OR Childcare OR Infant, Newborn OR Newborn Infant OR Newborn OR Neonate OR Infant OR Infant OR Child, Preschool OR Preschool OR Full Term Infant OR Neonatus OR Newly Born Baby OR Newly Born Child OR Newly Born Infant OR Pediatric OR Infant, Premature OR Preterm OR Premature OR Neonatal Prematurity OR Prematurita OR Child OR Child OR Children AND User Embracement OR Support OR Care OR Welcoming OR Embracement OR Child, Foster OR Foster Child OR Foster Youth OR Infant Care OR Child Care OR Child Day Care OR; Puericulture OR childcare OR Infant, Newborn OR Newborn Infant OR Newborn OR Neonate OR Infant OR Infant OR Child, Preschool OR Preschool OR Full Term Infant OR Neonatus OR Newly Born Baby OR Newly Born Child OR Newly Born Infant OR Pediatric OR Infant, Premature OR Preterm OR Premature OR Neonatal Prematurity OR Prematurita OR Child OR Child OR Children AND User Embracement OR Support OR Care OR Welcoming OR Embracement; OR Child, Foster OR Foster Child OR Foster Youth OR Infant Care OR Child Care OR Child Day Care; AND Transients and Migrants OR Migrant OR Nomad OR Nonmigrant OR Squatter OR Transient OR Refugee OR Expatriate OR Immigrant OR Transients and Migrants OR Migrant OR Nomad OR Nonmigrant OR Squatter OR Transient OR Refugee OR Expatriate OR Immigrant OR Transients and Migrants OR Migrant OR Nomad OR Nonmigrant OR Squatter OR Transient OR Refugee OR Expatriate OR Immigrant; AND Primary Health Care OR Primary Healthcare OR Primary Care OR Health Centers OR Health Center OR Health Posts OR Polyclinic OR Community Health Services OR Community Health Care OR Community Health Service OR Community Healthcare OR Primary Health Care OR Primary Healthcare OR Primary Care OR Health Centers OR Health Center OR Health Posts OR Polyclinic OR Community Health Services OR Community Health Care OR Community Health Service OR Community Healthcare OR Primary Health Care OR Primary Healthcare OR Primary Care OR Health Centers OR Health Center OR Health Posts OR Polyclinic OR Community Health Services OR Community Health Care OR Community Health Service OR Community Healthcare.	517
EMBASE/Elsevier	Puericulture:ti,ab,kw OR Childcare:ti,ab,kw OR Infant, Newborn’:ti,ab,kw OR Newborn Infant*’:ti,ab,kw OR Newborn*:ti,ab,kw OR Neonate*:ti,ab,kw OR ‘Infant’:ti,ab,kw OR Infant*:ti,ab,kw OR ‘Child, Preschool’:ti,ab,kw OR Preschool*:ti,ab,kw OR ‘Full term Infant’:ti,ab,kw OR Neonatus:ti,ab,kw OR ‘Newly Born Baby’:ti,ab,kw OR ‘Newly Born Child’:ti,ab,kw OR ‘Newly Born Infant’:ti,ab,kw OR Pediatric*:ti,ab,kw OR ‘Infant, Premature’:ti,ab,kw OR Preterm*:ti,ab,kw OR Premature*:ti,ab,kw OR ‘Neonatal Prematurity’:ti,ab,kw OR Prematurita*:ti,ab,kw OR ‘Child’:ti,ab,kw OR Child*:ti,ab,kw OR Children:ti,ab,kw) AND; ‘User Embracement’:ti,ab,kw OR Support:ti,ab,kw OR Care:ti,ab,kw OR Welcoming:ti,ab,kw OR Embracement:ti,ab,kw; OR ‘Child, Foster’:ti,ab,kw OR ‘Foster Child*’:ti,ab,kw OR ‘Foster Youth*’:ti,ab,kw OR ‘Infant Care’:ti,ab,kw OR ‘Child Care’:ti,ab,kw OR ‘Child Day Care’:ti,ab,kw; AND Transients:ti,ab,kw AND Migrants:ti,ab,kw OR Migrant*:ti,ab,kw OR Nomad*:ti,ab,kw OR Nonmigrant*:ti,ab,kw OR Squatter*:ti,ab,kw OR Transient*:ti,ab,kw OR Refugee*:ti,ab,kw OR Expatriate*:ti,ab,kw OR Immigrant*:ti,ab,kw; AND ‘Primary Health Care’:ti,ab,kw OR ‘Primary Healthcare’:ti,ab,kw OR ‘Primary Care’:ti,ab,kw OR ‘Health Centers’:ti,ab,kw OR ‘Health Center’:ti,ab,kw OR ‘Health Posts’:ti,ab,kw OR Polyclinic:ti,ab,kw OR ‘Community Health Services’:ti,ab,kw OR ‘Community Health Care’:ti,ab,kw OR ‘Community Health Service’:ti,ab,kw OR ‘Community Healthcare’:ti,ab,kw; AND [article]/lim OR [article in press]/lim OR [conference review]/lim OR [review]/lim OR [preprint]/lim.	568
PMC/NLM	Puericulture OR Childcare OR Infant, Newborn OR Newborn Infant OR Newborn OR Neonate OR Infant OR Infant OR Child, Preschool OR Preschool OR Full Term Infant OR Neonatus OR Newly Born Baby OR Newly Born Child OR Newly Born Infant OR Pediatric OR Infant, Premature OR Preterm OR Premature OR Neonatal Prematurity OR Prematurita OR Child OR Children AND User Embracement OR Support OR Care OR Welcoming OR Embracement OR Child, Foster OR Foster Child OR Foster Youth OR Infant Care OR Child Care OR Child Day Care AND Transients AND Migrants OR Migrant OR Nomad OR Nonmigrant OR Squatter OR Transient OR Refugee OR Expatriate OR Immigrant AND Primary Health Care OR Primary Healthcare OR Primary Care OR Health Centers OR Health Center OR Health Posts OR Polyclinic OR Community Health Services OR Community Health Care OR Community Health Service OR Community Healthcare.	473
PsycInfo/APA	Primary Health Care OR Primary Healthcare OR Primary Care OR Health Centers OR Health Center OR Health Posts OR Polyclinic OR Community Health Services OR Community Health Care OR Community Health Service OR Community Healthcare OR Primary Health Care OR Primary Healthcare OR Primary Care OR Health Centers OR Health Center OR Health Posts OR Polyclinic OR Community Health Services OR Community Health Care OR Community Health Service OR Community Healthcare; Primary Health Care OR Primary Healthcare OR Primary Care OR Health Centers OR Health Center OR Health Posts OR Polyclinic OR Community Health Services OR Community Health Care OR Community Health Service OR Community Healthcare AND Transients and Migrants OR Migrant OR Nomad OR Nonmigrant OR Squatter OR Transient OR Refugee OR Expatriate OR Immigrant OR Transients and Migrants OR Migrant OR Nomad OR Nonmigrant OR Squatter OR Transient OR Refugee OR Expatriate OR Immigrant OR Transients and Migrants OR Migrant OR Nomad OR Nonmigrant OR Squatter OR Transient OR Refugee OR Expatriate OR Immigrant AND Puericulture OR Childcare OR Infant, Newborn OR Newborn Infant OR Newborn OR Neonate OR Infant OR Infant OR Child, Preschool OR Preschool OR Full term Infant OR Neonatus OR Newly Born Baby OR Newly Born Child OR Newly Born Infant OR Pediatric OR Infant, Premature OR Preterm OR Premature OR Neonatal Prematurity OR Prematurita OR Child OR Child OR Children AND User Embracement OR Support OR Care OR Welcoming OR Embracement OR Child, Foster OR Foster Child OR Foster Youth OR Infant Care OR Child Care OR Child Day Care OR Puericulture OR Childcare OR Infant, Newborn OR Newborn Infant OR Newborn OR Neonate OR Infant OR Infant OR Child, Preschool OR Preschool OR Full Term Infant OR Neonatus OR Newly Born Baby OR Newly Born Child OR Newly Born Infant OR Pediatric OR Infant, Premature OR Preterm OR Premature OR Neonatal Prematurity OR Prematurita OR Child OR Child OR Children AND User Embracement OR Support OR Care OR Welcoming OR Embracement OR Child, Foster OR Foster Child OR Foster Youth OR Infant Care OR Child Care OR Child Day Care OR Puericulture OR Childcare OR Infant, Newborn OR Newborn Infant OR Newborn OR Neonate OR Infant OR Infant OR Child, Preschool OR Preschool OR Full Term Infant OR Neonatus OR Newly Born Baby OR Newly Born Child OR Newly Born Infant OR Pediatric OR Infant, Premature OR Preterm OR Premature OR Neonatal Prematurity OR Prematurita OR Child OR Children AND User Embracement OR Support OR Care OR Welcoming OR Embracement OR Child, Foster OR Foster Child OR Foster Youth OR Infant Care OR Child Care OR Child Day Care.	176
PubMed/NLM	Puericulture OR Childcare OR Infant, Newborn OR Newborn Infant OR Newborn OR Neonate OR Infant OR Infant OR Child, Preschool OR Preschool OR Full Term Infant OR Neonatus OR Newly Born Baby OR Newly Born Child OR Newly Born Infant OR Pediatric OR Infant, Premature OR Preterm OR Premature OR Neonatal Prematurity OR prematurita OR Child OR Children AND Embracement OR Support OR Care OR Welcoming OR Embracement OR Child, Foster OR Foster Child OR Foster Youth OR Infant Care OR Infant Care OR Child Care OR Child Day Care AND Transients and Migrants OR Transients and Migrants OR Migrant OR Nomad OR Nonmigrant OR Squatter OR Transient OR Refugee OR Expatriate OR Immigrant AND Primary Health Care OR Primary Health Care OR Primary Healthcare OR Primary Care OR Health Centers OR Health Center OR Health Posts OR Polyclinic OR Community Health Services OR Community Health Care OR Community Health Service OR Community Healthcare.	1.431
SciELO	Puericulture OR Childcare OR Infant, Newborn OR Newborn Infant OR Newborn OR Neonate OR Infant OR Infant OR Child, Preschool OR Preschool OR Full Term Infant OR Neonatus OR Newly Born Baby OR Newly Born Child OR Newly Born Infant OR Pediatric OR Infant, Premature OR Preterm OR Premature OR Neonatal Prematurity OR prematurita OR Child OR Children AND User Embracement OR Support OR Care OR Welcoming OR Embracement OR Child, Foster OR Foster Child OR Foster Youth OR Infant Care OR Child Care OR Child Day Care AND Transients and Migrants OR Migrant OR Nomad OR Nonmigrant OR Squatter OR Transient OR Refugee OR Expatriate OR Immigrant AND Primary Health Care OR Primary Healthcare OR Primary Care OR Health Centers OR Health Center OR Health Posts OR Polyclinic OR Community Health Services OR Community Health Care OR Community Health Service OR Community Healthcare.	28
Scopus/Elsevier	Puericulture OR Childcare OR Infant, Newborn OR Newborn Infant OR Newborn OR Neonate OR Infant OR Infant OR Child, Preschool OR Preschool OR Full Term Infant OR Neonatus OR Newly Born Baby OR Newly Born Child OR Newly Born Infant OR Pediatric OR Infant, Premature OR Preterm OR Premature OR Neonatal Prematurity OR Prematurita OR Child OR Children AND User Embracement OR Support OR Care OR Welcoming OR Embracement OR Child, Foster OR Foster Child OR Foster Youth OR Infant Care OR Child Care OR Child Day Care AND Transients and Migrants OR Migrant OR Nomad OR Nonmigrant OR Squatter OR Transient OR Refugee OR Expatriate OR Immigrant AND Primary Health Care OR Primary Healthcare OR Primary Care OR Health Centers OR Health Center OR Health Posts OR Polyclinic OR Community Health Services OR Community Health Care OR Community Health Service OR Community Healthcare.	1.457
*Web of Science*/Clarivate	Puericulture OR Childcare OR Infant, Newborn OR Newborn Infant OR Newborn OR Neonate OR Infant OR Infant OR Child, Preschool OR Preschool OR Full Term Infant OR Neonates OR Newly Born Baby OR Newly Born Child OR Newly Born Infant OR Pediatric OR Infant, Premature OR Preterm OR Premature OR Neonatal Prematurity OR Prematurita OR Child OR Child OR Children AND User Embracement OR Support OR Care OR Welcoming OR Embracement OR Child, Foster OR Foster Child OR Foster Youth OR Infant Care OR Child Care OR Child Day Care AND Transients and Migrants OR Migrant OR Nomad OR Nonmigrant OR Squatter OR Transient OR Refugee OR Expatriate OR Immigrant AND Primary Health Care OR Primary Healthcare OR Primary Care OR Health Centers OR Health Center OR Health Posts OR Polyclinic OR Community Health Services OR Community Health Care OR Community Health Service OR Community Healthcare.	687

As estratégias de busca listadas no quadro esquemático foram ajustadas às particularidades funcionais de cada base de dados, considerando os seus respectivos campos de indexação, a aplicação de operadores booleanos e os recursos específicos de recuperação da informação. Com o intuito de ampliar os achados para este estudo, os revisores definiram a estratégia de busca com o auxílio de uma bibliotecária com experiência em revisões de escopo, visando garantir a abrangência, a sensibilidade e a adequação dos descritores e palavras-chave utilizados. As estratégias foram validadas e adaptadas às especificidades de cada base de dados, respeitando os seus mecanismos de indexação e a recuperação da informação.

### Seleção dos Estudos

Os estudos recuperados nas buscas nas bases de dados foram importados para o EndNote 21 (*Clarivate Analytics*, PA, EUA), e as duplicatas foram removidas. As referências foram então exportadas para o *software Intelligent Systematic Review* (Rayyan) para gerenciamento da seleção. Na primeira fase, os estudos foram avaliados pelo título e resumo com base nos critérios de inclusão e, na segunda fase, pela análise do texto completo, que foi recuperado na íntegra. Na terceira e última etapa, todas as referências dos artigos incluídos foram revisadas manualmente para identificar as publicações adicionais que versam sobre o acolhimento da criança migrante na APS.

### Extração e Análise de Dados

O processo de seleção dos estudos foi conduzido por dois revisores independentes e de forma cega, com um terceiro revisor consultado em caso de divergências. Os motivos para a exclusão de fontes de evidência no texto completo que não atenderam aos critérios de inclusão foram registrados e relatados na revisão de escopo. Em seguida, os artigos selecionados foram analisados na íntegra, etapa que antecedeu a inclusão na amostra final.

Para a extração de dados, foram consideradas informações sobre autor, ano, país, tipo de estudo, nível de evidência estabelecidos pelo JBI^(11)^, participantes e práticas assistenciais de acolhimento direcionadas às crianças migrantes na APS. Essas evidências foram organizadas de maneira descritiva em um quadro-resumo (Quadro 2), desenvolvido pelos autores e adaptado às recomendações do manual do JBI^(8)^.

**Quadro 2:** Caracterização dos estudos incluídos na amostra final da revisão – Niterói, RJ, Brasil, 2026.

Código	Autor Ano País	Tipo de estudo Nível de evidência	Participantes	Práticas assistenciais
*A1^(51)^	Nascimento et al. 2024 Brasil	Estudo de ensaio clínico 2.b	População residente e circulante de áreas de fronteira brasileiras, incluindo crianças, brasileiros e estrangeiros (não descreve o quantitativo de participantes)	Vacinação
A2^(13)^	Ouzounidou et al. 2024 Grécia	Estudo transversal quantitativo e descritivo 4.b	54 médicos, 43 enfermeiros e 23 agentes comunitários de saúde	Vacinação Consultas
*A3^(14)^	Ayoub-Rodriguez et al. 2024 EUA	Artigo de opinião 5.b	Crianças migrantes (não descreve o quantitativo de participantes)	Consultas Administração de medicação
A4^(15)^	Reda et al. 2023 Noruega	Estudo de ensaio clínico 5.b	300 participantes (aproximadamente 50 pares pais/filhos de cada centro)	Acompanhamento odontológico Avaliação antropométrica
A5^(16)^	Yalçın et al. 2023 Turquia	Estudo descritivo 4.b	876 médicos∕ enfermeiros sírios (parteiras também foram consideradas nessa categoria)	Consultas Orientações em saúde
A6^(17)^	Kvamme e Voldner 2022 Noruega	Estudo descritivo e qualitativo 6.b	Sete enfermeiras de saúde pública	Consultas
A7^(18)^	Shon et al. 2020 Coreia do Sul	Estudo metodológico com teste de usabilidade 4.b	Oito enfermeiras comunitárias e 261 crianças	Consultas Verificação antropométrica Exames laboratoriais
A8^(19)^	Salami et al. 2020 Canadá	Estudo qualitativo e descritivo 6.b	17 pais e 33 mães	Consultas Vínculo
A9^(20)^	Zanatta et al. 2020 Brasil	Estudo exploratório, descritivo com abordagem qualitativa 6.b	Dez enfermeiros	Consultas Visitas domiciliares Avaliação antropométrica Orientações em saúde Acompanhamento nutricional
A10^(21)^	Ragavan et al. 2020 EUA	Estudo quantitativo e transversal 4.b	212 pais	Consultas
A11^(22)^	Shin et al. 2019 Coreia do Sul	Estudo descritivo e exploratório, com metodologia mista que incluiu revisão bibliográfica 4.b	16 moradores locais, 18 profissionais de saúde pública (enfermeiro e médico), oito líderes comunitários e 141 adulto de cada família	Consultas Visita domiciliar
A12^(23)^	Shin et al. 2019 Quirguistão	Estudo avaliativo retrospectivo de um projeto piloto 3.b	85 crianças	Avaliação antropométrica Visita domiciliar Exames laboratoriais Suplementação alimentar Consulta Triagem Orientações em saúde
A13^(24)^	Oldfield et al. 2019 EUA	Estudo qualitativo descritivo com análise de conteúdo 6.b	22 cuidadores	Consulta Orientações em saúde
A14^(25)^	Schrier et al. 2019 Europa	Estudo de revisão sistemática e comparativa 5.b	Crianças migrantes e refugiadas (não descreve o quantitativo de participantes)	Orientações em saúde Avaliação antropométrica Consultas Vacinação Exames laboratoriais
A15^(26)^	Pascual et al. 2018 Espanha	Estudo qualitativo e descritivo 6.b	17 profissionais de saúde e sete mães/pais	Consultas Orientações em saúde
A16^(27)^	Bull et al. 2018 EUA	Estudo projeto de melhoria de qualidade 3.b	68 pacientes registrados no local do projeto	Avaliação antropométrica Acompanhamento nutricional Acompanhamento do desenvolvimento Consultas
A17^(28)^	Carrasco-Sanz et al. 2018 Europa	Estudo quantitativo transversal 4.b	492 pediatras	Vacinação Consultas
A18^(29)^	Sami et al. 2018 Sudão do Sul	Estudo de caso 4.b	104 profissionais de saúde e sete gestores seniores	Consultas Encaminhamento para especialidades
A19^(30)^	Caballero et al. 2017 EUA	Estudo de revisão narrativa e de síntese crítica 5.b	Crianças e adolescentes migrantes	Acompanhamento psicológico Consultas
A20^(31)^	Arcury et al. 2016 EUA	Estudo quantitativo, observacional e transversal 4.b	221 mães de 221 crianças	Consultas Verificação antropométrica Acompanhamento nutricional
A21^(32)^	Fagerlund et al. 2016 Noruega	Estudo qualitativo e descritivo 6.b	26 enfermeiros de saúde pública	Acompanhamento nutricional Consultas Orientações em saúde Avaliação antropométrica
A22^(33)^	Cyril et al. 2016 Austrália	Estudo qualitativo e exploratório 6.b	59 prestadores de serviços envolvidos com a saúde e o bem-estar infantil	Acompanhamento nutricional Avaliação antropométrica Consultas
*A23^(34)^	Seery et al. 2015 EUA	Revisão de literatura 5.b	Crianças refugiadas (não descreve o quantitativo de participantes)	Avaliação de saúde Consultas Exames laboratoriais Acompanhamento nutricional Avaliação antropométrica Vacinação Acompanhamento psicológico
A24^(35)^	McMurray et al. 2014 Canadá	Estudo antes e depois 3.b	872 crianças/adultos	Consultas Encaminhamento para especialidades
*A25^(36)^	Measham et al. 2014 Canadá	Revisão de literatura 5.b	Crianças refugiadas e famílias refugiadas (não descreve o quantitativo de participantes)	Acompanhamento psicológico Encaminhamento para especialidades Consultas
*A26^(37)^	Huang e Au 2014 EUA	Estudo de relato de experiência 5.b	Famílias chinesas migrantes e crianças com necessidades especiais de saúde (não descreve o quantitativo de participantes)	Consultas Encaminhamento para especialidades
*A27^(38)^	Rousseau et al. 2013 Canadá	Estudo qualitativo exploratório 5.b	Crianças refugiadas (não descreve o quantitativo de participantes)	Acompanhamento social Acompanhamento psicológico Consultas
*A28^(39)^	Chilton et al. 2013 EUA	Revisão de literatura 5.b	Crianças migrantes e residentes em regiões de fronteira (não descreve o quantitativo de participantes)	Consultas Avaliação antropométrica Encaminhamento para especialidades
*A29^(40)^	Capella 2012 Espanha	Estudo teórico-conceitual de abordagem qualitativa, do tipo ensaio/reflexão bioética 5.b	Crianças de procedência internacional (não descreve o quantitativo de participantes)	Consultas Vínculo
*A30^(41)^	Woodland et al. 2010 Austrália	Desenvolvimento de um modelo baseado na literatura nacional e internacional e nos modelos de serviço atuais 5.b	Crianças refugiadas recém-chegadas e suas famílias (não descreve o quantitativo de participantes)	Triagem de saúde Consultas
*A31^(42)^	Vostanis 2010 Reino Unido	Estudo qualitativo, teórico-reflexivo, de revisão e discussão conceitual 5.b	Crianças e adolescentes pertencentes a grupos vulneráveis (não descreve o quantitativo de participantes)	Consultas Acompanhamento psicológico
A32^(43)^	Samarasinghe et al. 2010 Suécia	Estudo qualitativo, descritivo e exploratório 6.b	34 enfermeiros de saúde pública	Consultas Orientações em saúde Acompanhamento nutricional Visitas domiciliares
A33^(44)^	Swe e Ross 2010 EUA	Estudo exploratório 6.b	36 crianças/adolescentes/adultos/idosos	Triagem de saúde Vacinação Consultas Utilização de diversos serviços de saúde
A34^(45)^	Swingle et al. 2008 EUA	Estudo quanti-quali observacional 4.b	71 famílias	Consultas
*A35^(46)^	Paris e Bronson 2006 EUA	Estudo descritivo do tipo relato de experiência/descrição de programa de intervenção, com abordagem qualitativa 5.b	Mulheres migrantes e refugiadas consideradas de alto risco	Visitas domiciliares
A36^(47)^	Melvin 2006 EUA	Estudo descritivo, relato de experiência, avaliando um programa, observacional, longitudinal, utilizando comparação antes–depois 5.b	500 crianças	Serviços odontológicos Consultas
*A37^(52)^	Grant et al. 2005 Austrália	Revisão de literatura 5.b	Crianças, famílias e profissionais de saúde	Orientações em saúde
A38^(53)^	Ekezie et al. 2002 Alemanha	Estudo de revisão sistemática 4.b	104 estudos analisados	Vacinação Orientações em saúde
A39^(48)^	Clark 2002 EUA	Estudo qualitativo, do tipo etnográfico focalizado 6.b	25 mães	Consultas Vacinação
*A40^(49)^	Wilson et al. 2000 EUA	Estudo descritivo do tipo relato de experiência com abordagem quantitativa e qualitativa 6.b	Crianças hispânicas filhas de trabalhadores rurais migrantes	Intervenções de enfermagem Consultas Orientações em saúde Avaliação antropométrica Exames laboratoriais Encaminhamento para especialidade
A41^(54)^	Wilson et al. 2000 EUA	Estudo qualitativo, descritivo, exploratório 5.b	73 crianças	Exames laboratoriais Orientações em saúde
A42^(50)^	Doering et al. 1999 Canadá	Estudo descritivo do tipo relato de experiência 4.b	1.230 crianças	Consultas Exames laboratoriais

EUA – Estados Unidos da América;

*Os artigos não descrevem o quantitativo de participantes.

Foi realizada uma análise temática de abordagem indutiva, com categorias emergentes a partir dos dados. O processo de categorização foi orientado pelo referencial teórico-analítico de Bardin^(12)^. As categorias analíticas não foram previamente definidas, emergindo a partir da leitura e interpretação dos dados extraídos dos estudos incluídos.

O processo analítico ocorreu em três etapas: pré-análise, com leitura exaustiva dos artigos selecionados; exploração do material, mediante identificação e codificação dos temas recorrentes relacionados às práticas de acolhimento de crianças migrantes na APS; e tratamento e interpretação dos dados, com agrupamento dos códigos por similaridade temática e construção das categorias analíticas.

A análise foi conduzida de forma independente por dois revisores, sendo um terceiro consultado em situações de divergência. As discordâncias foram resolvidas por consenso, buscando assegurar o rigor metodológico, a rastreabilidade analítica e a redução de vieses interpretativos.

Esse processo possibilitou a construção de três categorias temáticas emergentes: “Ações clínicas e de acompanhamento na Atenção Primária à Saúde”; “Ações preventivas em saúde na Atenção Primária à Saúde”; e “Articulação da rede de saúde”.

## RESULTADOS

### Características Gerais dos Estudos

A busca nas bases de dados ocorreu em maio de 2024, quando foram identificados 5.389 documentos nas buscas realizadas nos bancos de dados Scopus (n = 1.457), PubMed (n = 1.431), *Web of Science* (n = 687), EMBASE (n = 568), EBSCO - CINAHL, Fonte Acadêmica e SociNDEX (n = 517), PMC (n = 473), PsycINFO (n = 176), BVS (n = 52), e SciELO (n = 28). Após a remoção de duplicatas, foi realizada uma seleção com base nos títulos, nos resumos e na leitura completa. Embora tenha sido feita uma busca manual nas listas de referências, nenhum novo estudo elegível foi identificado. Com base nos critérios de inclusão, foram selecionados 42 registros para a revisão de escopo (Figura 1).

**Figura 1:** Diagrama de fluxo *Preferred Reporting Items for Systematic reviews and Meta-Analyses extension for scoping reviews* do processo de seleção das publicações da revisão – Niterói, RJ, Brasil, 2026.

Os artigos analisados foram publicados entre 1999 e 2024, evidenciando um período amplo de produção científica sobre a temática. Os anos mais frequentes de publicação foram 2010, 2018, 2019 e 2020. Quanto à distribuição dos estudos por país, observou-se uma expressiva concentração da produção científica nos Estados Unidos, com 16 artigos contabilizados. Em seguida, o Canadá apresentou a segunda maior produção científica, com cinco artigos. Houve a predominância de pesquisas de natureza qualitativa, configurando-se como o tipo de estudo mais frequente nesta revisão de escopo. No total, 15 estudos adotaram abordagem qualitativa, incluindo delineamentos descritivos, exploratórios, interpretativos, etnográficos e análises de conteúdo.

Em relação ao nível de evidência dos 42 estudos incluídos, observou-se a predominância de estudos com baixo potencial de inferência causal, especialmente pesquisas qualitativas e descritivas, evidenciando que a produção científica sobre o acolhimento de crianças migrantes na APS ainda é majoritariamente exploratória e centrada na compreensão das experiências e práticas assistenciais. Para a questão de significado (*meaningfulness*), a maioria das evidências foi classificada no nível 3, composta por estudos qualitativos únicos que exploram as experiências de acolhimento. Já para a questão de eficácia (*effectiveness*), os estudos situaram-se majoritariamente no nível 4, representados por delineamentos observacionais descritivos e transversais. A concentração de evidências nesses níveis reflete a natureza do objeto de estudo, focado em contextos sociais e assistenciais complexos, nos quais ensaios clínicos controlados são menos frequentes.

As práticas assistenciais de acolhimento direcionadas às crianças migrantes foram estruturadas em três categorias analíticas. Para sintetizar esses achados, apresenta-se na Figura 2 um modelo conceitual que ilustra as categorias centrais, as suas dimensões e as inter-relações identificadas nesta revisão de escopo.

**Figura 2:** Diagrama conceitual das práticas assistenciais de acolhimento para crianças migrantes na Atenção Primária à Saúde.

### Ações Clínicas e de Acompanhamento na Atenção Primária à Saúde

Foram identificados 38 artigos que abordam as ações clínicas e de acompanhamento realizadas no âmbito da APS^(13,14,15,16,17,18,19,20,21,22,23,24,25,26,27,28,29,30,31,32,33,34,35,36,37,38,39,40,41,42,43,44,45,46,47,48,49,50)^. Essas ações incluíram consultas com profissionais de saúde de nível superior^(13,14,15,16,17,18,19,20,21,22,23,24,25,26,27,28,29,30,31,32,33,34,35,36,37,38,39,40,41,42,43,44,45,47,48,49,50)^, com foco na avaliação clínica, no acompanhamento do crescimento e desenvolvimento, no manejo de condições crônicas e agudas e no acompanhamento nutricional, odontológico e psicológico.

Também foram descritas práticas como as visitas domiciliares^(20,22,23,43,46)^, caracterizadas por contribuir para a compreensão das condições de vida das famílias de migrantes e para o fortalecimento do vínculo^(19,40)^ entre profissionais de saúde e usuários.

### Ações Preventivas em Saúde na Atenção Primária à Saúde

Nessa categoria, foram incluídos 21 artigos^(13,16,18,20,23,24,25,26,28,32,34,41,43,44,48,49,50,51,52,53,54)^, que relatam sobre as ações preventivas em saúde na APS, tendo como destaque a vacinação^(13,25,28,34,44,48,51,53)^ e a triagem em saúde^(23,41)^, especialmente entre crianças migrantes recém-chegadas. Durante esse primeiro momento no novo país, as crianças migrantes também realizam exames laboratoriais^(18,23,25,34,49,50,54)^ para rastrear doenças infecciosas.

Além disso, orientações em saúde^(16,20,23,24,25,32,43,49,52,53,54)^ foram descritas como estratégias transversais de acolhimento voltadas à promoção da saúde, à prevenção de agravos e ao apoio às famílias de migrantes recém-chegadas no uso dos serviços de saúde.

### Articulação da Rede de Saúde

Para essa última categoria, foram contabilizados seis artigos, que abordam sobre a articulação na rede de saúde^(29,35,36,37,39,49)^, que pôde ser identificada como um componente essencial do cuidado integral às crianças migrantes. Os estudos relataram o encaminhamento para serviços especializados, sociais e educacionais, evidenciando a necessidade de um cuidado integral e intersetorial para atender às múltiplas necessidades dessas crianças migrantes.

## DISCUSSÃO

As práticas assistenciais de acolhimento realizadas pelos profissionais de saúde para as crianças migrantes na APS, de modo geral, foram evidenciadas pelas ações de acompanhamento, de prevenção e de encaminhamento articulado na rede de saúde. Logo, o acolhimento não se caracteriza apenas como uma organização do fluxo de atendimento, mas como uma tecnologia utilizada para que se tenha, na APS, acesso à terapêutica^(55)^.

As ações clínicas e de acompanhamento foram as práticas mais frequentemente identificadas, incluindo consultas multiprofissionais, avaliação clínica, monitoramento do crescimento e desenvolvimento, avaliação antropométrica, acompanhamento nutricional e psicológico e visitas domiciliares. Esses resultados confirmam que a APS constitui a principal porta de entrada para o cuidado infantil em diversos sistemas de saúde, embora esse acesso possa ser limitado para populações migrantes em determinados contextos. Nesse sentido, a operacionalização desses serviços demanda o mapeamento sistemático dos desafios territoriais e dos fatores facilitadores que impulsionam a equidade no acesso^(56,57)^.

A predominância de consultas e do acompanhamento do crescimento e desenvolvimento reflete um cuidado centrado no modelo biomédico e programático de atenção à saúde da criança migrante, que prioriza o diagnóstico precoce de agravos e o acompanhamento longitudinal. Entretanto, quando se trata da população migrante, estudos apontam que essas populações enfrentam barreiras linguísticas, culturais e socioeconômicas que podem comprometer a efetividade dessas ações, ressaltando a necessidade de abordagens culturalmente sensíveis e centradas na família^(58)^.

As visitas domiciliares emergiram como estratégia relevante para o fortalecimento do vínculo e a compreensão do contexto familiar e social, sendo reconhecidas como ferramenta essencial na APS para identificação de determinantes sociais da saúde. Ao mesmo tempo, a literatura indica que essa prática é fundamental para compreender a população migrante e registrá-la no território. À medida que os profissionais identificam esse grupo como parte permanente ou transitória da área, torna-se necessário adotar estratégias que promovam seu acolhimento e integração ao fluxo de atendimento da unidade^(59)^.

Destaca-se a importância da APS e do seu papel central na saúde da criança, pois é a principal porta de entrada dos usuários no SUS. A APS compreende ações de saúde nos âmbitos individual, familiar e coletivo, incluindo promoção, prevenção, proteção, diagnóstico, tratamento, reabilitação, redução de danos, cuidados paliativos e vigilância em saúde, as quais devem ser desenvolvidas por meio de práticas de cuidado integral e de uma gestão qualificada^(56)^.

As ações realizadas pelos profissionais com a finalidade de prevenção, particularmente vacinação, triagem em saúde, exames laboratoriais e orientações em saúde, foram amplamente descritas nos estudos incluídos. A vacinação foi destacada como uma das principais estratégias de acolhimento. Estudos apontam que a imunização é a principal estratégia de prevenção e controle das doenças imunopreveníveis, pois se trata de uma ação essencial de saúde pública^(60)^.

No Brasil, a vacinação é caracterizada por ser um ato constitucional, até mesmo para os recém-nascidos filhos de migrantes, pois se trata de um indicador nacional. Além disso, fronteira, conta-se com o suporte de postos que realizam a imunização. Nesse primeiro contato, são feitas a triagem de saúde e a vacinação de povos migrantes que adentram o país^(61)^.

No contexto brasileiro e latino-americano, a aplicabilidade desses achados deve ser analisada com cautela, considerando as especificidades dos sistemas de saúde, as desigualdades sociais e os fluxos migratórios regionais. Embora o SUS preconize o acesso universal, barreiras estruturais, organizacionais e socioculturais ainda podem dificultar o acesso e a continuidade do cuidado para populações migrantes. Dessa forma, estratégias descritas na literatura internacional podem não ser diretamente transferíveis, demandando adaptações que considerem as desigualdades estruturais, a organização do SUS e as especificidades dos fluxos migratórios na região.

As orientações em saúde e as atividades educativas foram descritas como estratégias transversais voltadas à promoção da saúde e ao apoio às crianças migrantes no uso dos serviços de saúde. Essas orientações devem considerar, prioritariamente, os aspectos culturais e linguísticos, bem como a trajetória sociocultural dessa população, sem preconceitos ou estigmas que possam interferir na prestação do cuidado^(59)^.

O contato entre o profissional e a criança deve ocorrer de forma positiva entre ambos, mas estudos apontam uma recorrência negativa. Também são raras as reflexões críticas que considerem as necessidades individuais, culturais e socioeconômicas dos migrantes. Por isso, a relação entre profissionais e migrantes tende a ser, em alguns casos, conflituosa e com pouca resolução das demandas e dos problemas de saúde informados^(62)^.

Logo, para que se tenha a resolução das demandas trazidas, é necessário repensar os processos de trabalho com base no reconhecimento e no diagnóstico situacional do território, delineando estratégias e considerando as dificuldades que restringem o acesso das crianças migrantes. Além disso, faz-se fundamental assegurar e qualificar o atendimento aos usuários migrantes em todas as esferas de assistência à saúde dessa população^(59)^.

A articulação na rede de saúde foi a categoria menos frequente nos estudos incluídos, sendo relatada principalmente por meio de encaminhamentos para serviços especializados, sociais e educacionais. Esse achado evidencia que embora seja reconhecida como essencial para o cuidado integral, a integração intersetorial ainda é pouco descrita e operacionalizada na literatura sobre crianças migrantes na APS. Estudos apontam que o apoio intersetorial, quando ajustado às necessidades das famílias de migrantes, pode melhorar a promoção da saúde das crianças migrantes^(62)^.

Diante desse cenário, a intersetorialidade pode ser compreendida como uma estratégia fundamental para superar a fragmentação dos saberes e das políticas sociais por meio da articulação entre diferentes setores. Essa integração possibilita o desenvolvimento de ações conjuntas e complementares, com potencial para enfrentar iniquidades em saúde e promover a melhoria das condições de vida das populações^(63)^.

A relação entre a saúde e a migração apresenta uma dinâmica complexa para quem vive todo esse processo. Embora não seja uma regra, parte da população migrante pode estar exposta a agravos à saúde e a condições precárias de vida^(59)^, fazendo-se necessário que os profissionais de saúde da APS estejam dispostos a realizar o acolhimento.

Embora os achados desta revisão apontem para a centralidade da APS no cuidado às crianças migrantes, é importante considerar as limitações dos estudos incluídos. Observou-se a predominância de estudos qualitativos, descritivos e observacionais, classificados majoritariamente em níveis moderados e baixos de evidência, segundo os critérios do JBI. Esse achado demonstra que a produção científica acerca do acolhimento de crianças migrantes na APS ainda se concentra na compreensão de experiências, percepções e práticas assistenciais, havendo escassez de estudos analíticos ou de intervenção capazes de produzir evidências com maior potencial de generalização e inferência causal.

Ademais, a possível presença de viés de publicação deve ser considerada, uma vez que estudos que reportam experiências exitosas de acolhimento tendem a ser mais frequentemente publicados em detrimento de evidências que revelem fragilidades ou falhas na assistência.

A complexidade do processo migratório e os seus impactos na saúde infantil reforçam a necessidade de qualificação das práticas de acolhimento na APS, incorporando abordagens que considerem os determinantes sociais, culturais e estruturais. Contudo, permanecem lacunas importantes na literatura quanto à efetividade dessas estratégias, especialmente em países de baixa e média renda. Nesse sentido, destaca-se a necessidade de estudos futuros com maior rigor metodológico e de políticas públicas que promovam a equidade no acesso e na qualidade do cuidado ofertado às crianças migrantes.

Embora a literatura internacional apresente modelos consolidados de clínicas dedicadas a refugiados no hemisfério norte, a aplicabilidade desses achados no cenário do SUS e em outros contextos da América Latina exige uma análise criteriosa. Diferentemente de sistemas baseados em seguros ou centros de recepção isolados, o acolhimento no Brasil deve ser operacionalizado dentro da rede universal da APS, prezando pela não fragmentação do cuidado. Assim, as práticas de acolhimento em territórios fronteiriços, como o de Roraima, devem focar no fortalecimento de Unidades Básicas de Saúde como fontes habituais de cuidado, integrando a vigilância epidemiológica à sensibilidade cultural necessária para o atendimento das famílias venezuelanas.

## CONCLUSÃO

A presente revisão de escopo mapeou as práticas assistenciais de acolhimento destinadas às crianças migrantes na APS, evidenciando que acolher tem sido operacionalizado, predominantemente, por meio de ações clínicas e de acompanhamento, ações preventivas e articulação na rede de serviços.

Os achados reforçam o papel central da APS como porta de entrada preferencial para o cuidado infantil e como coordenadora da rede de atenção, evidenciando o acolhimento como uma tecnologia relacional e organizacional fundamental para garantir o acesso e a continuidade do cuidado à criança migrante. A articulação intersetorial com os serviços de assistência social, educação e proteção social foi a categoria menos descrita nos estudos, indicando fragilidades na coordenação do cuidado e na implementação de políticas públicas integradas.

Portanto, há uma necessidade clara de estudos futuros que abordem estratégias de acolhimento culturalmente competentes, intervenções intersetoriais estruturadas e a avaliação da efetividade das práticas assistenciais direcionadas às crianças migrantes na APS. Dessa forma, recomenda-se o desenvolvimento de pesquisas futuras que aprofundem a compreensão das necessidades dessa população e subsidiem a formulação de políticas e práticas de cuidado mais integradas, equitativas e centradas na criança e na família.

## Data Availability

Todo o conjunto de dados que dá suporte aos resultados deste estudo foi publicado no próprio artigo.

# Translation: Prácticas de atención para la acogida de niños migrantes en la Atención Primaria de Salud: revisión de alcance

**DOI:** 10.1590/1980-220X-REEUSP-2026-0121es

**Authors:** Ana Beatriz Oliveira Costa, Lanna Jeniffer Silva Rodrigues, Mônica Letícia Martins Franco, Paulo Sérgio da Silva, Audrey Vidal Pereira, Liliane Faria da Silva

**Affiliations:** 1 Universidade Federal Fluminense, Programa Acadêmico em Ciências do Cuidado em Saúde, Niterói, RJ, Brasil.; 2 Universidade Federal de Lavras, Faculdade de Ciências da Saúde, Lavras, MG, Brasil.

**Keywords:** Acogimiento, Atención Primaria de Salud, Atención Integral de Salud, Migrantes

## Abstract

**Objetivo:**

Mapear y sintetizar la evidencia disponible sobre las prácticas de atención a niños migrantes en Atención Primaria de Salud.

**Método:**

Revisión del alcance realizada según las recomendaciones de JBI y descrita mediante la lista de verificación PRISMA-ScR. Se realizaron búsquedas en las siguientes bases de datos: MEDLINE/PubMed, CINAHL, Academic Search Premier, Academic Search Ultimate (EBSCO), Scopus, EMBASE/Elsevier, APA PsycInfo (BVS), LILACS, BDENF, Index Psychology – Periodicals y SciELO.

**Resultados:**

Se incluyeron 42 estudios publicados entre 1999 y 2024. La evidencia se clasificó en acciones clínicas y de seguimiento en Atención Primaria de Salud, acciones de salud preventiva en Atención Primaria de Salud y articulación de la red de salud. Los estudios incluidos presentaron heterogeneidad en cuanto a diseños metodológicos, contextos de implementación y características de las poblaciones investigadas.

**Conclusión:**

La atención se ha centrado en acciones clínicas, preventivas y de coordinación en red, reafirmando la Atención Primaria de Salud como principal punto de acceso al cuidado infantil. Sin embargo, esto pone de manifiesto la necesidad de futuros estudios que aborden estrategias de atención culturalmente competentes, intervenciones intersectoriales estructuradas y la evaluación de la eficacia de estas prácticas.

## INTRODUCCIÓN

En primer lugar, conviene contextualizar que el fenómeno migratorio consiste en el desplazamiento de la población entre diferentes regiones. En cuanto a su alcance espacial, la migración puede clasificarse como interna, cuando se limita a los límites territoriales de un Estado soberano, o internacional, cuando implica el cruce de fronteras nacionales y el establecimiento en un país extranjero^(1)^. De hecho, el proceso migratorio influye directamente en la salud del migrante, y el proceso de adaptación al nuevo lugar no siempre es positivo. La persona migrante puede volverse vulnerable debido a su situación social y, en algunos casos, enfrentar el aislamiento y el alejamiento familiar, lo que aumenta la probabilidad de sufrir estigma, discriminación y prejuicios^(2)^.

Al ingresar a un nuevo país, las personas migrantes se enfrentan a barreras para acceder a los servicios de salud, entre ellas la falta de seguro médico, el temor a la deportación, la exclusión de los programas gubernamentales y las diferencias lingüísticas y culturales^(3)^. Específicamente, los niños en situación de movilidad presentan una elevada vulnerabilidad social y están expuestos a riesgos graves, como la inseguridad alimentaria, la trata de personas, la explotación sexual y la negligencia en la atención de la salud. En este sentido, el proceso migratorio —que comprende el desplazamiento y el cruce de fronteras bajo represión, además de la compleja readaptación sociocultural— somete a la niñez a eventos estresantes con repercusiones profundas y duraderas en su salud mental^(4)^.

Ante estas consideraciones y teniendo en cuenta las particularidades de la población infantil migrante, la Atención Primaria de Salud (APS) es una estrategia central en el ámbito de la salud pública, sobre todo porque permite monitorear de manera longitudinal el crecimiento y el desarrollo de los niños, estimula la participación comunitaria en los servicios de salud y contribuye a la promoción del bienestar de las poblaciones. Además, es indispensable señalar que la APS ha contribuido al fortalecimiento de las relaciones entre la comunidad y los actores del sistema de salud, lo que favorece el empoderamiento a nivel local y regional del Sistema Único de Salud (SUS)^(5)^.

En esta línea, las necesidades en salud de la población infantil migrante en el marco de la APS pueden reconocerse a través de la acogida en salud, entendida como un mecanismo que reconoce al usuario y sus necesidades en salud como legítimas y singulares, sustentando la dimensión relacional y humana del proceso de atención. El acto de acoger puede involucrar otras herramientas de atención, como la escucha calificada, que contribuye a la construcción de vínculos y a una comprensión más profunda de las necesidades migratorias^(6)^.

En Brasil, a lo largo de los años, la salud infantil ha presentado avances en la organización de la atención en el ámbito de la APS. Esta mejora en la calidad de la atención es resultado de la consolidación de los derechos legales de los niños y de la evolución de las políticas públicas de salud en el contexto nacional. En la APS, el niño recibe servicios de atención basados en los principios del derecho universal a la vida, la equidad, la integralidad de la atención, la humanización, la atención y la gestión participativa^(7)^.

Con base en estas consideraciones preliminares, en abril de 2024 se realizó una búsqueda preliminar en el *International Prospective Register of Systematic Reviews* (PROSPERO) y en las principales bases de datos, sin que se identificara ninguna revisión de alcance o sistemática, publicada o en curso, relacionada con las particularidades de la población infantil migrante. En el contexto presentado, se habla poco y de manera detallada sobre las prácticas asistenciales de acogida para niños migrantes que brindan los profesionales de la salud en el ámbito de la APS.

Desde esta perspectiva, se llevó a cabo una revisión de alcance (en inglés, *scoping review*) con el objetivo de mapear y sintetizar la evidencia disponible sobre las prácticas asistenciales de acogida dirigidas a los niños migrantes en la APS. A partir de ello, se buscó identificar brechas, promover la equidad en el acceso a la salud, respaldar las políticas públicas y mejorar la calidad de la atención a los niños migrantes en la atención primaria, con el fin de garantizar una atención culturalmente sensible a las necesidades de la población infantil migrante.

## MÉTODO

### Diseño del Estudio

Se trata de una revisión de alcance, elaborada de acuerdo con el marco teórico proporcionado por el JBI^(8)^. Este tipo de revisión tiene como objetivo mapear la evidencia científica principal y las limitaciones de un tema específico disponibles en la literatura, siguiendo las recomendaciones propuestas por los *Preferred Reporting Items for Systematic reviews and Meta-Analyses extension for Scoping Reviews* (PRISMA-ScR), a partir de una extensión para revisiones de alcance^(9)^. Durante la fase exploratoria de la revisión, se buscó verificar la ausencia de informes de investigación o protocolos de revisión recientes que abordaran una temática similar a la del presente estudio. Posteriormente, el protocolo de esta revisión se registró en la plataforma *Open Science Framework* (https://doi.org/10.17605/OSF.IO/ZSTNX).

Dado que se trata de una revisión de alcance, cuyo objetivo es mapear y caracterizar la evidencia disponible en la literatura, no se llevó a cabo una evaluación crítica formal de la calidad metodológica de los estudios incluidos. Esta decisión está en consonancia con las recomendaciones del JBI, que no considera obligatoria la evaluación de la calidad en las revisiones de alcance, especialmente cuando el enfoque se centra en la amplitud de la evidencia y la identificación de brechas de conocimiento.

### Identificación de la Pregunta de Investigación

Para la formulación de la pregunta de investigación, se adoptó el método Población, Concepto y Contexto (PCC), una estrategia que ayuda a identificar los temas clave y que se recomienda para la realización de revisiones de alcance. Por lo tanto, el desarrollo de este estudio partió de la siguiente pregunta: ¿cuáles son las prácticas de acogida para niños migrantes de 0 a 10 años en la atención primaria de salud?

Se incluyeron estudios que tuvieran como población objetivo a niños de 0 a 10 años, con un enfoque específico en los migrantes. En este estudio, se consideran migrantes a las personas que se desplazan de su lugar de residencia habitual, ya sea dentro de un país o a través de una frontera internacional, de manera temporal o permanente^(10)^.

### Criterios de Selección

Esta revisión de alcance abarcó estudios experimentales y cuasi-experimentales, incluyendo ensayos clínicos aleatorizados, ensayos clínicos no aleatorizados, estudios de antes y después y estudios de series temporales interrumpidas. Además, se incluyeron estudios observacionales analíticos de tipo cohorte prospectivos y retrospectivos, estudios de caso-control y estudios transversales. También se incluyeron estudios observacionales descriptivos, como series de casos, informes de casos individuales y estudios transversales descriptivos. Asimismo, se incluyeron estudios cualitativos que abarcaban diferentes enfoques, como la fenomenología, la teoría fundamentada, la etnografía, la descripción cualitativa, la investigación-acción y la investigación feminista. Se consideraron las revisiones de cualquier tipo que cumplieran con los criterios de inclusión y que estuvieran en consonancia con la pregunta de investigación. Por último, también se incluyeron textos y artículos de opinión en esta revisión de alcance. Se excluyeron los resúmenes y las cartas al editor.

Dado que se trata de una revisión de alcance, cuyo objetivo es mapear y caracterizar la evidencia disponible en la literatura, no se realizó una evaluación crítica formal de la calidad metodológica de los estudios incluidos. Esta decisión está en consonancia con las recomendaciones del JBI, que no considera obligatoria la evaluación de la calidad en las revisiones de alcance, especialmente cuando el enfoque se centra en la amplitud de la evidencia y la identificación de brechas de conocimiento.

### Estrategia de Búsqueda

La estrategia de búsqueda se llevó a cabo con el objetivo de localizar estudios publicados y no publicados. Inicialmente, se realizó una búsqueda preliminar considerando los términos de los Descriptores en Ciencias de la Salud, los *Medical Subject Headings* y los *Embase Subject Headings*, que se combinaron mediante los operadores booleanos *OR* y *AND* en MEDLINE/PubMed, OSF, PROSPERO, *JBI Synthesis* y *Cochrane Library*. El objetivo era identificar protocolos, revisiones y artículos primarios, así como nuevas palabras clave y términos de indexación estandarizados para una estrategia integral.

De manera secundaria, los términos identificados conformaron la estrategia de búsqueda definitiva, la cual se adaptó a las bases de datos y fuentes de información definidas para la revisión, a saber: MEDLINE/PubMed; *Cumulative Index to Nursing and Allied Health Literature* (CINAHL); *Academic Search Premier* y *Academic Source* de EBSCO; Scopus; EMBASE de Elsevier; y APA *PsycInfo*. También se consultaron las bases de datos del Portal Regional de la Biblioteca Virtual en Salud (BVS): Literatura Latinoamericana y del Caribe en ciencias de la salud (LILACS), Base de Datos de Enfermería (BDENF) e *Index Psychology – Periodicals*, así como el portal de revistas *Scientific Electronic Library Online* (SciELO). Se incluyeron estudios sin restricciones de idioma ni límite temporal.

El Cuadro 1 presenta las estrategias de búsqueda adoptadas en cada base de datos, organizadas según el acrónimo PCC, con descriptores y palabras clave dispuestos por bloques, explicación de la lógica booleana y número de registros encontrados.

**Cuadro 1:** Estrategia de búsqueda realizada el 10 de mayo de 2024 para cada base de datos – Niterói, RJ, Brasil, 2024.

Bases de datos	Estrategias de búsqueda	Registros encontrados (n)
LILACSPLUS/BVS	Migrantes OR Migrante OR Nômade OR Nômades OR Nomadismo OR Não-migrante OR Não-migrantes OR Posseiro OR Posseiros OR Transeunte OR Transeuntes OR Nómada OR Nómadas OR Nomadismo OR Ocupante OR Migratorio OR Population de Passage et Migrants OR Migrants OR Nomade OR Population de passage OR Population en Transit OR Population en Transit et Migrants OR Sédentaires OR Squatters OR Squatteurs OR Squatteuses OR Transients and Migrants OR Migrant OR Nomad OR Nonmigrant OR Squatter OR Transien; AND Criança OR Infantil OR Infancia OR Infant, Newborn OR Newborn Infant OR Newborn OR Neonate OR Infant OR Child, Preschool OR Preschool OR Full Term Infant OR Neonatus OR Newly Born Baby OR Newly Born Child OR Newly Born Infant OR Pediatric OR Infant, Premature OR Preterm OR Premature OR Neonatal Prematurity OR Prematurita OR Recem-Nascido OR Neonato OR Lactente OR Prematuro OR Infancia OR Cuidado do Lactente OR Cuidado del Lactante OR Atención del Lactante OR Soins du Nourrisson OR Soins au Nouveau-né et au Nourrisson OR Soins aux Nouveau-nés et aux Nourrissons OR Soins des Nourrissons OR Soins Infantiles OR Cuidado da Criança OR Cuidado Infantil OR puericultura OR Cuidado del Niño OR Cuidado Infantil OR Puericultura OR Soins de L’enfant OR Soins aux Enfants OR Soins des Enfants OR Infant Care OR Child Care OR Child Day Care OR puericulture; AND Acolhimento OR User Embracement OR Acogimiento OR Adoption par l’Utilisateur OR Amparo OR Assistência OR Abrigamento OR Cuidado OR Support OR Care OR Welcoming OR Apoyo OR Asistencia OR Bienvenida OR Integración OR Adoption par l’Utilisateur OR Accueil OR Soutien OR Assistance OR Bienvenue OR Intégration; OR Criança Acolhida OR Criança Bem-Vinda OR Criança em Regime de Acolhimento OR Crianças Acolhidas OR Crianças Bem-Vindas OR Crianças em Regime de Acolhimento OR Jovem Acolhido OR Jovem Bem-Vindo OR Jovens Acolhidos OR Jovens Bem-Vindos OR Niño Acogido OR Joven Acogido OR Jóvenes en Régimen de Acogida OR Niña Acogida OR Niños Acogidos OR Enfant Placé en Famille d’accueil OR Enfant Accueilli OR Enfants accueillis OR Enfants Placés en Familles D’accueil OR Jeune Accueilli OR Jeune Placé en Famille D’accueil OR Child, Foster OR Foster Child OR Foster Children OR Foster Youth OR Foster Youths; AND Atenção Primária à Saúde OR Atención Primaria de Salud OR Atendimento Básico OR Atendimento Primário OR Atenção Básica OR Atenção Básica de Saúde OR Atenção Básica à Saúde OR Atenção Primária OR Atenção Primária de Saúde OR Atenção Primária em Saúde OR Cuidados Primários OR Cuidados Primários de Saúde OR Cuidados Primários à Saúde OR Cuidados de Saúde Primários OR Centros de Saúde OR Centros de Salud OR Centro de Saúde OR Posto de Assistência Médica OR Posto de Saúde OR Postos de Saúde OR Unidade Básica de Saúde OR Serviços Básicos de Saúde OR Servicios Básicos de Salud OR Prestações Básicas de Saúde OR Unidade de Saúde OR Unidade de Serviço OR Prestaciones Básicas de Salud OR Centro de Salud OR Postas Médicas OR Puestos Médicos OR Puestos de Salud OR Primary Health Care OR Primary Healthcare OR Primary Care OR Health Centers OR Health Center OR Health Posts OR Basic Health Services OR Basic Health Care Provisions OR Community Health Services OR Community Health Care OR Community Health Service OR Community Healthcare OR Assistência à Saúde Comunitária OR Assistência Médica Comunitária OR Cuidados Médicos Comunitários OR Atención de la Salud Comunitaria OR Atención Médica Comunitaria OR Services de Santé Communautaires OR Services de Santé Communautaire OR Services de Santé de Proximité OR Soins de Santé Communautaires OR Soins de Santé de Proximité; AND Instance: lilacsplus.	52
CINAHL e SocINDEX/EBSCO	Puericulture OR Childcare OR Infant, Newborn OR Newborn Infant OR Newborn OR Neonate OR Infant OR Infant OR Child, Preschool OR Preschool OR Full Term Infant OR Neonatus OR Newly Born Baby OR Newly Born Child OR Newly Born Infant OR Pediatric OR Infant, Premature OR Preterm OR Premature OR Neonatal Prematurity OR prematurita OR Child OR Child OR Children; AND User Embracement OR support OR care OR welcoming OR Embracement; OR Child, Foster OR Foster Child OR Foster Youth OR Infant Care OR Child Care OR Child Day Care; OR Puericulture OR Childcare OR Infant, Newborn OR Newborn Infant OR Newborn OR Neonate OR Infant OR Infant OR Child, Preschool OR Preschool OR Full Term Infant OR Neonatus OR Newly Born Baby OR Newly Born Child OR Newly Born Infant OR Pediatric OR Infant, Premature OR Preterm OR Premature OR Neonatal Prematurity OR Prematurita OR Child OR Child OR Children AND User Embracement OR Support OR Care OR Welcoming OR Embracement OR Child, Foster OR Foster Child OR Foster Youth OR Infant Care OR Child Care OR Child Day Care OR; Puericulture OR childcare OR Infant, Newborn OR Newborn Infant OR Newborn OR Neonate OR Infant OR Infant OR Child, Preschool OR Preschool OR Full Term Infant OR Neonatus OR Newly Born Baby OR Newly Born Child OR Newly Born Infant OR Pediatric OR Infant, Premature OR Preterm OR Premature OR Neonatal Prematurity OR Prematurita OR Child OR Child OR Children AND User Embracement OR Support OR Care OR Welcoming OR Embracement; OR Child, Foster OR Foster Child OR Foster Youth OR Infant Care OR Child Care OR Child Day Care; AND Transients and Migrants OR Migrant OR Nomad OR Nonmigrant OR Squatter OR Transient OR Refugee OR Expatriate OR Immigrant OR Transients and Migrants OR Migrant OR Nomad OR Nonmigrant OR Squatter OR Transient OR Refugee OR Expatriate OR Immigrant OR Transients and Migrants OR Migrant OR Nomad OR Nonmigrant OR Squatter OR Transient OR Refugee OR Expatriate OR Immigrant; AND Primary Health Care OR Primary Healthcare OR Primary Care OR Health Centers OR Health Center OR Health Posts OR Polyclinic OR Community Health Services OR Community Health Care OR Community Health Service OR Community Healthcare OR Primary Health Care OR Primary Healthcare OR Primary Care OR Health Centers OR Health Center OR Health Posts OR Polyclinic OR Community Health Services OR Community Health Care OR Community Health Service OR Community Healthcare OR Primary Health Care OR Primary Healthcare OR Primary Care OR Health Centers OR Health Center OR Health Posts OR Polyclinic OR Community Health Services OR Community Health Care OR Community Health Service OR Community Healthcare.	517
EMBASE/Elsevier	Puericulture:ti,ab,kw OR Childcare:ti,ab,kw OR Infant, Newborn’:ti,ab,kw OR Newborn Infant*’:ti,ab,kw OR Newborn*:ti,ab,kw OR Neonate*:ti,ab,kw OR ‘Infant’:ti,ab,kw OR Infant*:ti,ab,kw OR ‘Child, Preschool’:ti,ab,kw OR Preschool*:ti,ab,kw OR ‘Full term Infant’:ti,ab,kw OR Neonatus:ti,ab,kw OR ‘Newly Born Baby’:ti,ab,kw OR ‘Newly Born Child’:ti,ab,kw OR ‘Newly Born Infant’:ti,ab,kw OR Pediatric*:ti,ab,kw OR ‘Infant, Premature’:ti,ab,kw OR Preterm*:ti,ab,kw OR Premature*:ti,ab,kw OR ‘Neonatal Prematurity’:ti,ab,kw OR Prematurita*:ti,ab,kw OR ‘Child’:ti,ab,kw OR Child*:ti,ab,kw OR Children:ti,ab,kw) AND; ‘User Embracement’:ti,ab,kw OR Support:ti,ab,kw OR Care:ti,ab,kw OR Welcoming:ti,ab,kw OR Embracement:ti,ab,kw; OR ‘Child, Foster’:ti,ab,kw OR ‘Foster Child*’:ti,ab,kw OR ‘Foster Youth*’:ti,ab,kw OR ‘Infant Care’:ti,ab,kw OR ‘Child Care’:ti,ab,kw OR ‘Child Day Care’:ti,ab,kw; AND Transients:ti,ab,kw AND Migrants:ti,ab,kw OR Migrant*:ti,ab,kw OR Nomad*:ti,ab,kw OR Nonmigrant*:ti,ab,kw OR Squatter*:ti,ab,kw OR Transient*:ti,ab,kw OR Refugee*:ti,ab,kw OR Expatriate*:ti,ab,kw OR Immigrant*:ti,ab,kw; AND ‘Primary Health Care’:ti,ab,kw OR ‘Primary Healthcare’:ti,ab,kw OR ‘Primary Care’:ti,ab,kw OR ‘Health Centers’:ti,ab,kw OR ‘Health Center’:ti,ab,kw OR ‘Health Posts’:ti,ab,kw OR Polyclinic:ti,ab,kw OR ‘Community Health Services’:ti,ab,kw OR ‘Community Health Care’:ti,ab,kw OR ‘Community Health Service’:ti,ab,kw OR ‘Community Healthcare’:ti,ab,kw; AND [article]/lim OR [article in press]/lim OR [conference review]/lim OR [review]/lim OR [preprint]/lim.	568
PMC/NLM	Puericulture OR Childcare OR Infant, Newborn OR Newborn Infant OR Newborn OR Neonate OR Infant OR Infant OR Child, Preschool OR Preschool OR Full Term Infant OR Neonatus OR Newly Born Baby OR Newly Born Child OR Newly Born Infant OR Pediatric OR Infant, Premature OR Preterm OR Premature OR Neonatal Prematurity OR Prematurita OR Child OR Children AND User Embracement OR Support OR Care OR Welcoming OR Embracement OR Child, Foster OR Foster Child OR Foster Youth OR Infant Care OR Child Care OR Child Day Care AND Transients AND Migrants OR Migrant OR Nomad OR Nonmigrant OR Squatter OR Transient OR Refugee OR Expatriate OR Immigrant AND Primary Health Care OR Primary Healthcare OR Primary Care OR Health Centers OR Health Center OR Health Posts OR Polyclinic OR Community Health Services OR Community Health Care OR Community Health Service OR Community Healthcare.	473
PsycInfo/APA	Primary Health Care OR Primary Healthcare OR Primary Care OR Health Centers OR Health Center OR Health Posts OR Polyclinic OR Community Health Services OR Community Health Care OR Community Health Service OR Community Healthcare OR Primary Health Care OR Primary Healthcare OR Primary Care OR Health Centers OR Health Center OR Health Posts OR Polyclinic OR Community Health Services OR Community Health Care OR Community Health Service OR Community Healthcare; Primary Health Care OR Primary Healthcare OR Primary Care OR Health Centers OR Health Center OR Health Posts OR Polyclinic OR Community Health Services OR Community Health Care OR Community Health Service OR Community Healthcare AND Transients and Migrants OR Migrant OR Nomad OR Nonmigrant OR Squatter OR Transient OR Refugee OR Expatriate OR Immigrant OR Transients and Migrants OR Migrant OR Nomad OR Nonmigrant OR Squatter OR Transient OR Refugee OR Expatriate OR Immigrant OR Transients and Migrants OR Migrant OR Nomad OR Nonmigrant OR Squatter OR Transient OR Refugee OR Expatriate OR Immigrant AND Puericulture OR Childcare OR Infant, Newborn OR Newborn Infant OR Newborn OR Neonate OR Infant OR Infant OR Child, Preschool OR Preschool OR Full term Infant OR Neonatus OR Newly Born Baby OR Newly Born Child OR Newly Born Infant OR Pediatric OR Infant, Premature OR Preterm OR Premature OR Neonatal Prematurity OR Prematurita OR Child OR Child OR Children AND User Embracement OR Support OR Care OR Welcoming OR Embracement OR Child, Foster OR Foster Child OR Foster Youth OR Infant Care OR Child Care OR Child Day Care OR Puericulture OR Childcare OR Infant, Newborn OR Newborn Infant OR Newborn OR Neonate OR Infant OR Infant OR Child, Preschool OR Preschool OR Full Term Infant OR Neonatus OR Newly Born Baby OR Newly Born Child OR Newly Born Infant OR Pediatric OR Infant, Premature OR Preterm OR Premature OR Neonatal Prematurity OR Prematurita OR Child OR Child OR Children AND User Embracement OR Support OR Care OR Welcoming OR Embracement OR Child, Foster OR Foster Child OR Foster Youth OR Infant Care OR Child Care OR Child Day Care OR Puericulture OR Childcare OR Infant, Newborn OR Newborn Infant OR Newborn OR Neonate OR Infant OR Infant OR Child, Preschool OR Preschool OR Full Term Infant OR Neonatus OR Newly Born Baby OR Newly Born Child OR Newly Born Infant OR Pediatric OR Infant, Premature OR Preterm OR Premature OR Neonatal Prematurity OR Prematurita OR Child OR Children AND User Embracement OR Support OR Care OR Welcoming OR Embracement OR Child, Foster OR Foster Child OR Foster Youth OR Infant Care OR Child Care OR Child Day Care.	176
PubMed/NLM	Puericulture OR Childcare OR Infant, Newborn OR Newborn Infant OR Newborn OR Neonate OR Infant OR Infant OR Child, Preschool OR Preschool OR Full Term Infant OR Neonatus OR Newly Born Baby OR Newly Born Child OR Newly Born Infant OR Pediatric OR Infant, Premature OR Preterm OR Premature OR Neonatal Prematurity OR prematurita OR Child OR Children AND Embracement OR Support OR Care OR Welcoming OR Embracement OR Child, Foster OR Foster Child OR Foster Youth OR Infant Care OR Infant Care OR Child Care OR Child Day Care AND Transients and Migrants OR Transients and Migrants OR Migrant OR Nomad OR Nonmigrant OR Squatter OR Transient OR Refugee OR Expatriate OR Immigrant AND Primary Health Care OR Primary Health Care OR Primary Healthcare OR Primary Care OR Health Centers OR Health Center OR Health Posts OR Polyclinic OR Community Health Services OR Community Health Care OR Community Health Service OR Community Healthcare.	1.431
SciELO	Puericulture OR Childcare OR Infant, Newborn OR Newborn Infant OR Newborn OR Neonate OR Infant OR Infant OR Child, Preschool OR Preschool OR Full Term Infant OR Neonatus OR Newly Born Baby OR Newly Born Child OR Newly Born Infant OR Pediatric OR Infant, Premature OR Preterm OR Premature OR Neonatal Prematurity OR prematurita OR Child OR Children AND User Embracement OR Support OR Care OR Welcoming OR Embracement OR Child, Foster OR Foster Child OR Foster Youth OR Infant Care OR Child Care OR Child Day Care AND Transients and Migrants OR Migrant OR Nomad OR Nonmigrant OR Squatter OR Transient OR Refugee OR Expatriate OR Immigrant AND Primary Health Care OR Primary Healthcare OR Primary Care OR Health Centers OR Health Center OR Health Posts OR Polyclinic OR Community Health Services OR Community Health Care OR Community Health Service OR Community Healthcare.	28
Scopus/Elsevier	Puericulture OR Childcare OR Infant, Newborn OR Newborn Infant OR Newborn OR Neonate OR Infant OR Infant OR Child, Preschool OR Preschool OR Full Term Infant OR Neonatus OR Newly Born Baby OR Newly Born Child OR Newly Born Infant OR Pediatric OR Infant, Premature OR Preterm OR Premature OR Neonatal Prematurity OR Prematurita OR Child OR Children AND User Embracement OR Support OR Care OR Welcoming OR Embracement OR Child, Foster OR Foster Child OR Foster Youth OR Infant Care OR Child Care OR Child Day Care AND Transients and Migrants OR Migrant OR Nomad OR Nonmigrant OR Squatter OR Transient OR Refugee OR Expatriate OR Immigrant AND Primary Health Care OR Primary Healthcare OR Primary Care OR Health Centers OR Health Center OR Health Posts OR Polyclinic OR Community Health Services OR Community Health Care OR Community Health Service OR Community Healthcare.	1.457
*Web of Science*/Clarivate	Puericulture OR Childcare OR Infant, Newborn OR Newborn Infant OR Newborn OR Neonate OR Infant OR Infant OR Child, Preschool OR Preschool OR Full Term Infant OR Neonates OR Newly Born Baby OR Newly Born Child OR Newly Born Infant OR Pediatric OR Infant, Premature OR Preterm OR Premature OR Neonatal Prematurity OR Prematurita OR Child OR Child OR Children AND User Embracement OR Support OR Care OR Welcoming OR Embracement OR Child, Foster OR Foster Child OR Foster Youth OR Infant Care OR Child Care OR Child Day Care AND Transients and Migrants OR Migrant OR Nomad OR Nonmigrant OR Squatter OR Transient OR Refugee OR Expatriate OR Immigrant AND Primary Health Care OR Primary Healthcare OR Primary Care OR Health Centers OR Health Center OR Health Posts OR Polyclinic OR Community Health Services OR Community Health Care OR Community Health Service OR Community Healthcare.	687

Las estrategias de búsqueda enumeradas en el cuadro esquemático se adaptaron a las particularidades funcionales de cada base de datos, tomando en cuenta sus respectivos campos de indexación, la aplicación de operadores booleanos y los recursos específicos para la recuperación de información. Con el fin de ampliar los hallazgos de este estudio, los revisores definieron la estrategia de búsqueda con la ayuda de una bibliotecaria con experiencia en revisiones de alcance, con el objetivo de garantizar la amplitud, la sensibilidad y la adecuación de los descriptores y las palabras clave utilizados. Las estrategias se validaron y se adaptaron a las especificidades de cada base de datos, respetando sus mecanismos de indexación y de recuperación de información.

### Selección de los Estudios

Los estudios recuperados en las búsquedas en las bases de datos se importaron a EndNote 21 (*Clarivate Analytics*, PA, EE. UU.), y se eliminaron los duplicados. A continuación, las referencias se exportaron al *software Intelligent Systematic Review* (Rayyan) para gestionar la selección. En la primera fase, los estudios se evaluaron por su título y resumen con base en los criterios de inclusión y, en la segunda fase, mediante el análisis del texto completo, que se recuperó en su totalidad. En la tercera y última etapa, todas las referencias de los artículos incluidos se revisaron manualmente para identificar publicaciones adicionales que traten sobre la atención a los niños migrantes en la atención primaria de salud.

### Extracción y Análisis de Datos

El proceso de selección de los estudios fue llevado a cabo por dos revisores independientes y de manera ciega, con un tercer revisor al que se consultó en caso de discrepancias. Los motivos para la exclusión de fuentes de evidencia en el texto completo que no cumplieron con los criterios de inclusión se registraron y se incluyeron en la revisión del alcance. Posteriormente, se analizaron íntegramente los artículos seleccionados, etapa que precedió a su inclusión en la muestra final.

Para la extracción de datos, se tomó en cuenta información sobre el autor, el año, el país, el tipo de estudio, el nivel de evidencia establecido por el JBI^(11)^, los participantes y las prácticas asistenciales de acogida dirigidas a los niños migrantes en la APS. Estas evidencias se organizaron de manera descriptiva en un cuadro resumen (Cuadro 2), elaborado por los autores y adaptado a las recomendaciones del manual del JBI^(8)^.

**Cuadro 2:** Caracterización de los estudios incluidos en la muestra final de la revisión – Niterói, RJ, Brasil, 2026.

Código	Autor Año País	Tipo de estudio Nivel de evidencia	Participantes	Prácticas asistenciales
*A1^(51)^	Nascimento et al. 2024 Brasil	Estudio de ensayo clínico 2.b	Población residente y de paso en zonas fronterizas brasileñas, incluyendo niños, brasileños y extranjeros (no se especifica el número de participantes)	Vacunación
A2^(13)^	Ouzounidou et al. 2024 Grecia	Estudio transversal cuantitativo y descriptivo 4.b	54 médicos, 43 enfermeros y 23 agentes comunitarios de salud	Vacunación Consultas
*A3^(14)^	Ayoub-Rodríguez et al. 2024 EE. UU.	Artículo de opinión 5.b	Niños migrantes (no se especifica el número de participantes)	Consultas Administración de medicación
A4^(15)^	Reda et al. 2023 Noruega	Estudio clínico 5.b	300 participantes (aproximadamente 50 parejas de padres e hijos de cada centro)	Seguimiento odontológico Evaluación antropométrica
A5^(16)^	Yalçın et al. 2023 Turquía	Estudio descriptivo 4.b	876 médicos y enfermeros sirios (las comadronas también se incluyeron en esta categoría)	Consultas Orientación sanitaria
A6^(17)^	Kvamme y Voldner 2022 Noruega	Estudio descriptivo y cualitativo 6.b	Siete enfermeras de salud pública	Consultas
A7^(18)^	Shon et al. 2020 Corea del Sur	Estudio metodológico con prueba de usabilidad 4.b	Ocho enfermeras comunitarias y 261 niños	Consultas Medidas antropométricas Análisis de laboratorio
A8^(19)^	Salami et al. 2020 Canadá	Estudio cualitativo y descriptivo 6.b	17 padres y 33 madres	Consultas Vínculo
A9^(20)^	Zanatta et al. 2020 Brasil	Estudio exploratorio y descriptivo con enfoque cualitativo 6.b	Diez enfermeros	Consultas Visitas a domicilio Evaluación antropométrica Orientación sanitaria Seguimiento nutricional
A10^(21)^	Ragavan et al. 2020 EE. UU.	Estudio cuantitativo y transversal 4.b	212 padres	Consultas
A11^(22)^	Shin et al. 2019 Corea del Sur	Estudio descriptivo y exploratorio, con metodología mixta que incluyó una revisión bibliográfica 4.b	16 residentes locales, 18 profesionales de la salud pública (enfermeros y médicos), ocho líderes comunitarios y 141 adultos de cada familia	Consultas Visita a domicilio
A12^(23)^	Shin et al. 2019 Kirguistán	Estudio evaluativo retrospectivo de un proyecto piloto 3.b	85 niños	Evaluación antropométrica Visita a domicilio Análisis de laboratorio Suplementación alimentaria Consulta Cribado Orientación sanitaria
A13^(24)^	Oldfield et al. 2019 EE. UU.	Estudio cualitativo descriptivo con análisis de contenido 6.b	22 cuidadores	Consulta Orientación sanitaria
A14^(25)^	Schrier et al. 2019 Europa	Estudio de revisión sistemática y comparativo 5.b	Niños migrantes y refugiados (no se indica el número de participantes)	Orientaciones en materia de salud Evaluación antropométrica Consultas Vacunación Análisis de laboratorio
A15^(26)^	Pascual et al. 2018 España	Estudio cualitativo y descriptivo 6.b	17 profesionales sanitarios y siete madres/padres	Consultas Orientaciones sanitarias
A16^(27)^	Bull et al. 2018 EE. UU.	Estudio de un proyecto de mejora de la calidad 3.b	68 pacientes registrados en el lugar del proyecto	Evaluación antropométrica Seguimiento nutricional Seguimiento del desarrollo Consultas
A17^(28)^	Carrasco-Sanz et al. 2018 Europa	Estudio cuantitativo transversal 4.b	492 pediatras	Vacunación Consultas
A18^(29)^	Sami et al. 2018 Sudán del Sur	Estudio de caso 4.b	104 profesionales sanitarios y siete altos cargos	Consultas Derivaciones a especialidades
A19^(30)^	Caballero et al. 2017 EE. UU.	Estudio de revisión narrativa y síntesis crítica 5.b	Niños y adolescentes migrantes	Acompañamiento psicológico Consultas
A20^(31)^	Arcury et al. 2016 EE. UU.	Estudio cuantitativo, observacional y transversal 4.b	221 madres de 221 niños	Consultas Evaluación antropométrica Seguimiento nutricional
A21^(32)^	Fagerlund et al. 2016 Noruega	Estudio cualitativo y descriptivo 6.b	26 enfermeros de salud pública	Seguimiento nutricional Consultas Orientación sanitaria Evaluación antropométrica
A22^(33)^	Cyril et al. 2016 Australia	Estudio cualitativo y exploratorio 6.b	59 profesionales dedicados a la salud y el bienestar infantil	Seguimiento nutricional Evaluación antropométrica Consultas
*A23^(34)^	Seery et al. 2015 EE. UU.	Revisión bibliográfica 5.b	Niños refugiados (no se especifica el número de participantes)	Evaluación de salud Consultas Análisis de laboratorio Seguimiento nutricional Evaluación antropométrica Vacunación Acompañamiento psicológico
A24^(35)^	McMurray et al. 2014 Canadá	Estudio de antes y después 3.b	872 niños/adultos	Consultas Derivación a especialistas
*A25^(36)^	Measham et al. 2014 Canadá	Revisión bibliográfica 5.b	Niños y familias refugiadas (no se especifica el número de participantes)	Acompañamiento psicológico Derivación a especialistas Consultas
*A26^(37)^	Huang y Au 2014 EE. UU.	Estudio de caso 5.b	Familias chinas migrantes y niños con necesidades especiales de salud (no se indica el número de participantes)	Consultas Derivación a especialistas
*A27^(38)^	Rousseau et al. 2013 Canadá	Estudio cualitativo exploratorio 5.b	Niños refugiados (no se especifica el número de participantes)	Acompañamiento social Acompañamiento psicológico Consultas
*A28^(39)^	Chilton et al. 2013 EE. UU.	Revisión bibliográfica 5.b	Niños migrantes y residentes en regiones fronterizas (no se indica el número de participantes)	Consultas Evaluación antropométrica Derivación a especialistas
*A29^(40)^	Capella 2012 España	Estudio teórico-conceptual de enfoque cualitativo, del tipo ensayo/reflexión bioética 5.b	Niños de origen internacional (no se especifica el número de participantes)	Consultas Enlace
*A30^(41)^	Woodland et al. 2010 Australia	Desarrollo de un modelo basado en la literatura nacional y en en los modelos de servicio actuales 5.b	Niños refugiados recién llegados y sus familias (no se especifica el número de participantes)	Evaluación médica Consultas
*A31^(42)^	Vostanis 2010 Reino Unido	Estudio cualitativo, teórico-reflexivo, de revisión y debate conceptual 5.b	Niños y adolescentes pertenecientes a grupos vulnerables (no se especifica el número de participantes)	Consultas Acompañamiento psicológico
A32^(43)^	Samarasinghe et al. 2010 Suecia	Estudio cualitativo, descriptivo y exploratorio 6.b	34 enfermeros de salud pública	Consultas Orientación sanitaria Seguimiento nutricional Visitas a domicilio
A33^(44)^	Swe y Ross 2010 EE. UU.	Estudio exploratorio 6.b	36 niños/adolescentes/adultos/personas mayores	Cribado sanitario Vacunación Consultas Uso de diversos servicios sanitarios
A34^(45)^	Swingle et al. 2008 EE. UU.	Estudio observacional cuantitativo-cualitativo 4.b	71 familias	Consultas
*A35^(46)^	Paris y Bronson 2006 EE. UU.	Estudio descriptivo del tipo relato de experiencia/descripción de un programa de intervención, con enfoque cualitativo 5.b	Mujeres migrantes y refugiadas consideradas de alto riesgo	Visitas domiciliarias
A36^(47)^	Melvin 2006 EE. UU.	Estudio descriptivo, relato de experiencia, evaluación de un programa, e o observacional, longitudinal, con comparación antes-después 5.b	500 niños	Servicios odontológicos Consultas
*A37^(52)^	Grant et al. 2005 Australia	Revisión bibliográfica 5.b	Niños, familias y profesionales sanitarios	Orientaciones sanitarias
A38^(53)^	Ekezie et al. 2002 Alemania	Estudio de revisión sistemática 4.b	104 estudios analizados	Vacunación Directrices sanitarias
A39^(48)^	Clark 2002 EE. UU.	Estudio cualitativo, de tipo etnográfico focalizado 6.b	25 madres	Consultas Vacunación
*A40^(49)^	Wilson et al. 2000 EE. UU.	Estudio descriptivo de tipo «relato de experiencia» con un enfoque cuantitativo y cualitativo 6.b	Niñas hispanas hijas de trabajadores rurales migrantes	Intervenciones de enfermería Consultas Orientaciones sobre salud Evaluación antropométrica Análisis clínicos Derivación a especialistas
A41^(54)^	Wilson et al. 2000 EE. UU.	Estudio cualitativo, descriptivo, exploratorio 5.b	73 niños	Análisis de laboratorio Orientación sanitaria
A42^(50)^	Doering et al. 1999 Canadá	Estudio descriptivo de tipo informe de experiencia 4.b	1.230 niños	Consultas Análisis de laboratorio

EE. UU. – Estados Unidos de América;

*Los artículos no indican el número de participantes.

Se llevó a cabo un análisis temático de enfoque inductivo, con categorías emergentes a partir de los datos. El proceso de categorización se orientó por el marco teórico-analítico de Bardin^(12)^. Las categorías analíticas no se definieron previamente, sino que surgieron a partir de la lectura e interpretación de los datos extraídos de los estudios incluidos.

El proceso analítico se desarrolló en tres etapas: preanálisis, con lectura exhaustiva de los artículos seleccionados; exploración del material, mediante la identificación y codificación de los temas recurrentes relacionados con las prácticas de atención a los niños migrantes en la APS; y tratamiento e interpretación de los datos, con agrupación de los códigos por similitud temática y construcción de las categorías analíticas.

El análisis fue realizado de manera independiente por dos revisores, y se consultó a un tercero en situaciones de divergencia. Las discrepancias se resolvieron por consenso, con el fin de garantizar el rigor metodológico, la trazabilidad analítica y la reducción de sesgos interpretativos.

Este proceso permitió la construcción de tres categorías temáticas emergentes: «Acciones clínicas y de seguimiento en la Atención Primaria de Salud»; «Acciones preventivas en salud en la Atención Primaria de Salud»; y «Articulación de la red de salud».

## RESULTADOS

### Características Generales de los Estudios

La búsqueda en las bases de datos se llevó a cabo en mayo de 2024, cuando se identificaron 5 389 documentos en las búsquedas realizadas en las bases de datos Scopus (n = 1 457), PubMed (n = 1 431), *Web of Science* (n = 687), EMBASE (n = 568), EBSCO - CINAHL, Fonte Acadêmica y SociNDEX (n = 517), PMC (n = 473), PsycINFO (n = 176), BVS (n = 52) y SciELO (n = 28). Tras eliminar los duplicados, se llevó a cabo una selección basada en los títulos, los resúmenes y la lectura completa. Aunque se realizó una búsqueda manual en las listas de referencias, no se identificó ningún nuevo estudio elegible. Con base en los criterios de inclusión, se seleccionaron 42 registros para la revisión de alcance (Figura 1).

**Figura 1:** Diagrama de flujo *Preferred Reporting Items for Systematic reviews and Meta-Analyses extension for scoping reviews* del proceso de selección de las publicaciones de la revisión – Niterói, RJ, Brasil, 2026.

Los artículos analizados se publicaron entre 1999 y 2024, lo que pone de manifiesto un amplio período de producción científica sobre el tema. Los años con mayor frecuencia de publicación fueron 2010, 2018, 2019 y 2020. En cuanto a la distribución de los estudios por país, se observó una concentración significativa de la producción científica en Estados Unidos, con 16 artículos registrados. A continuación, Canadá presentó la segunda mayor producción científica, con cinco artículos. Predominaron las investigaciones de naturaleza cualitativa, que se configuraron como el tipo de estudio más frecuente en esta revisión de alcance. En total, 15 estudios adoptaron un enfoque cualitativo, incluyendo diseños descriptivos, exploratorios, interpretativos, etnográficos y análisis de contenido.

En cuanto al nivel de evidencia de los 42 estudios incluidos, se observó un predominio de estudios con bajo potencial de inferencia causal, especialmente investigaciones cualitativas y descriptivas, lo que pone de manifiesto que la producción científica sobre la acogida de niños migrantes en la atención primaria de salud (APS) sigue siendo mayoritariamente exploratoria y centrada en la comprensión de las experiencias y prácticas asistenciales. En cuanto a la cuestión de la relevancia (*meaningfulness*), la mayoría de la evidencia se clasificó en el nivel 3, compuesta por estudios cualitativos únicos que exploran las experiencias de acogida. En cuanto a la cuestión de la eficacia (*effectiveness*), los estudios se situaron mayoritariamente en el nivel 4, representados por diseños observacionales descriptivos y transversales. La concentración de evidencia en estos niveles refleja la naturaleza del objeto de estudio, enfocado en contextos sociales y asistenciales complejos, en los que los ensayos clínicos controlados son menos frecuentes.

Las prácticas asistenciales de acogida dirigidas a los niños migrantes se estructuraron en tres categorías analíticas. Para sintetizar estos hallazgos, en la Figura 2 se presenta un modelo conceptual que ilustra las categorías centrales, sus dimensiones y las interrelaciones identificadas en esta revisión de alcance.

**Figura 2:** Diagrama conceptual de las prácticas asistenciales de acogida para niños migrantes en la Atención Primaria de Salud.

### Acciones Clínicas y de Seguimiento en la Atención Primaria de Salud

Se identificaron 38 artículos que abordan las acciones clínicas y de seguimiento realizadas en el ámbito de la APS^(13,14,15,16,17,18,19,20,21,22,23,24,25,26,27,28,29,30,31,32,33,34,35,36,37,38,39,40,41,42,43,44,45,46,47,48,49,50)^. Estas acciones incluyeron consultas con profesionales de la salud con formación superior^(13,14,15,16,17,18,19,20,21,22,23,24,25,26,27,28,29,30,31,32,33,34,35,36,37,38,39,40,41,42,43,44,45,47,48,49,50)^, centradas en la evaluación clínica, el seguimiento del crecimiento y el desarrollo, el manejo de afecciones crónicas y agudas, y el seguimiento nutricional, odontológico y psicológico.

También se describieron prácticas como las visitas a domicilio^(20,22,23,43,46)^, que se caracterizan por contribuir a la comprensión de las condiciones de vida de las familias migrantes y al fortalecimiento del vínculo^(19,40)^ entre los profesionales de la salud y los usuarios.

### Acciones Preventivas en Salud en la Atención Primaria de Salud

En esta categoría se incluyeron 21 artículos^(13,16,18,20,23,24,25,26,28,32,34,41,43,44,48,49,50,51,52,53,54)^, que informan sobre las medidas preventivas en salud en la APS, entre las que destacan la vacunación^(13,25,28,34,44,48,51,53)^ y la evaluación de salud^(23,41)^, especialmente entre los niños migrantes recién llegados. Durante esta primera etapa en el nuevo país, los niños migrantes también se someten a exámenes de laboratorio^(18,23,25,34,49,50,54)^ para detectar enfermedades infecciosas.

Además, las orientaciones en salud^(16,20,23,24,25,32,43,49,52,53,54)^ se describieron como estrategias transversales de acogida orientadas a la promoción de la salud, la prevención de afecciones y el apoyo a las familias de migrantes recién llegadas en el uso de los servicios de salud.

### Coordinacion de la Red de Salud

Para esta última categoría, se contabilizaron seis artículos que abordan la coordinación en la red de salud^(29,35,36,37,39,49)^, la cual se identificó como un componente esencial de la atención integral a los niños migrantes. Los estudios informaron sobre la derivación a servicios especializados, sociales y educativos, lo que pone de manifiesto la necesidad de una atención integral e intersectorial para satisfacer las múltiples necesidades de estos niños migrantes.

## DISCUSIÓN

Las prácticas asistenciales de atención inicial que los profesionales de la salud brindan a los niños migrantes en la APS, en general, se manifestaron a través de acciones de seguimiento, prevención y derivación coordinadas dentro de la red de salud. Por lo tanto, la atención inicial no se caracteriza únicamente como una organización del flujo de atención, sino como una estrategia utilizada para garantizar, en la APS, el acceso a la terapia^(55)^.

Las acciones clínicas y de seguimiento fueron las prácticas identificadas con mayor frecuencia, incluyendo consultas multiprofesionales, evaluación clínica, monitoreo del crecimiento y desarrollo, evaluación antropométrica, seguimiento nutricional y psicológico, y visitas a domicilio. Estos resultados confirman que la APS constituye la principal puerta de entrada a la atención infantil en diversos sistemas de salud, aunque dicho acceso puede ser limitado para las poblaciones migrantes en determinados contextos. En este sentido, la puesta en marcha de estos servicios exige un mapeo sistemático de los desafíos territoriales y de los factores facilitadores que impulsan la equidad en el acceso^(56,57)^.

El predominio de las consultas y del seguimiento del crecimiento y el desarrollo refleja una atención centrada en el modelo biomédico y programático de atención de salud del niño migrante, que prioriza el diagnóstico precoz de afecciones y el seguimiento longitudinal. Sin embargo, en lo que respecta a la población migrante, los estudios señalan que estas poblaciones enfrentan barreras lingüísticas, culturales y socioeconómicas que pueden comprometer la efectividad de estas acciones, lo que resalta la necesidad de enfoques culturalmente sensibles y centrados en la familia^(58)^.

Las visitas a domicilio se han revelado como una estrategia relevante para fortalecer el vínculo y comprender el contexto familiar y social, y se reconocen como una herramienta esencial en la atención primaria de salud (APS) para identificar los determinantes sociales de la salud. Al mismo tiempo, la literatura indica que esta práctica es fundamental para comprender a la población migrante y registrarla en el territorio. A medida que los profesionales identifican a este grupo como parte permanente o transitoria de la zona, se hace necesario adoptar estrategias que promuevan su acogida e integración en el flujo de atención de la unidad^(59)^.

Cabe destacar la importancia de la APS y su papel central en la salud infantil, ya que es la principal puerta de entrada de los usuarios al SUS. La APS abarca acciones de salud en los ámbitos individual, familiar y colectivo, incluyendo la promoción, la prevención, la protección, el diagnóstico, el tratamiento, la rehabilitación, la reducción de daños, los cuidados paliativos y la vigilancia en salud, las cuales deben desarrollarse mediante prácticas de atención integral y una gestión calificada^(56)^.

Las acciones realizadas por los profesionales con fines de prevención, en particular la vacunación, la evaluación en salud, los exámenes de laboratorio y la orientación en salud, se describieron ampliamente en los estudios incluidos. La vacunación se destacó como una de las principales estrategias de atención inicial. Los estudios señalan que la inmunización es la principal estrategia de prevención y control de las enfermedades prevenibles mediante vacunas, ya que se trata de una acción esencial de salud pública^(60)^.

En Brasil, la vacunación se caracteriza por ser un acto constitucional, incluso para los recién nacidos hijos de migrantes, ya que se trata de un indicador nacional. Además, en la frontera, se cuenta con el apoyo de puestos que realizan la inmunización. En este primer contacto, se lleva a cabo el cribado de salud y la vacunación de las personas migrantes que ingresan al país^(61)^.

En el contexto brasileño y latinoamericano, la aplicabilidad de estos hallazgos debe analizarse con cautela, teniendo en cuenta las particularidades de los sistemas de salud, las desigualdades sociales y los flujos migratorios regionales. Aunque el SUS promueve el acceso universal, las barreras estructurales, organizativas y socioculturales aún pueden dificultar el acceso y la continuidad de la atención para las poblaciones migrantes. Por lo tanto, es posible que las estrategias descritas en la literatura internacional no sean directamente transferibles, lo que exige adaptaciones que tomen en cuenta las desigualdades estructurales, la organización del SUS y las particularidades de los flujos migratorios en la región.

Las orientaciones en salud y las actividades educativas se han descrito como estrategias transversales orientadas a la promoción de la salud y al apoyo a los niños migrantes en el uso de los servicios de salud. Estas orientaciones deben considerar, de manera prioritaria, los aspectos culturales y lingüísticos, así como la trayectoria sociocultural de esta población, sin prejuicios ni estigmas que puedan interferir en la prestación de la atención^(59)^.

El contacto entre el profesional y la niña o el niño debe ser positivo para ambos, pero los estudios señalan una recurrencia de experiencias negativas. También son escasas las reflexiones críticas que tomen en cuenta las necesidades individuales, culturales y socioeconómicas de los migrantes. Por ello, la relación entre los profesionales y los migrantes tiende a ser, en algunos casos, conflictiva y con escasa resolución de las demandas y los problemas de salud reportados^(62)^.

Por lo tanto, para que se resuelvan las demandas planteadas, es necesario replantear los procesos de trabajo basándose en el reconocimiento y el diagnóstico situacional del territorio, definiendo estrategias y tomando en cuenta las dificultades que restringen el acceso de los niños migrantes. Además, es fundamental garantizar y mejorar la calidad de la atención a los usuarios migrantes en todos los ámbitos de la asistencia en salud de esta población^(59)^.

La coordinación en la red de salud fue la categoría menos frecuente en los estudios incluidos, y se reportó principalmente a través de derivaciones a servicios especializados, sociales y educativos. Este hallazgo pone de manifiesto que, aunque se reconoce como esencial para la atención integral, la integración intersectorial aún se describe y se pone en práctica de manera limitada en la literatura sobre niños migrantes en la APS. Los estudios indican que el apoyo intersectorial, cuando se adapta a las necesidades de las familias migrantes, puede mejorar la promoción de la salud de los niños migrantes^(62)^.

Ante este panorama, la intersectorialidad puede entenderse como una estrategia fundamental para superar la fragmentación de los conocimientos y las políticas sociales mediante la articulación entre diferentes sectores. Esta integración permite el desarrollo de acciones conjuntas y complementarias, con potencial para enfrentar las desigualdades en salud y promover la mejora de las condiciones de vida de las poblaciones^(63)^.

La relación entre la salud y la migración presenta una dinámica compleja para quienes viven todo este proceso. Aunque no es una regla, parte de la población migrante puede estar expuesta a problemas de salud y a condiciones de vida precarias^(59)^, lo que hace necesario que los profesionales de salud de la APS estén dispuestos a brindar atención de acogida.

Aunque los hallazgos de esta revisión apuntan a la importancia central de la APS en la atención a los niños migrantes, es importante considerar las limitaciones de los estudios incluidos. Se observó un predominio de estudios cualitativos, descriptivos y observacionales, clasificados en su mayoría en niveles moderados y bajos de evidencia, según los criterios del JBI. Este hallazgo demuestra que la producción científica sobre la atención a los niños migrantes en la APS aún se centra en la comprensión de experiencias, percepciones y prácticas asistenciales, y que hay una escasez de estudios analíticos o de intervención capaces de generar evidencia con mayor potencial de generalización e inferencia causal.

Además, se debe considerar la posible presencia de un sesgo de publicación, ya que los estudios que informan sobre experiencias exitosas de atención tienden a publicarse con mayor frecuencia, en detrimento de la evidencia que revela debilidades o fallas en la atención.

La complejidad del proceso migratorio y sus impactos en la salud infantil refuerzan la necesidad de mejorar las prácticas de acogida en la atención primaria de salud, incorporando enfoques que consideren los determinantes sociales, culturales y estructurales. Sin embargo, persisten importantes lagunas en la literatura en cuanto a la efectividad de estas estrategias, especialmente en países de ingresos bajos y medios. En este sentido, se destaca la necesidad de estudios futuros con mayor rigor metodológico y de políticas públicas que promuevan la equidad en el acceso y en la calidad de la atención que se brinda a los niños migrantes.

Aunque la literatura internacional presenta modelos consolidados de clínicas dedicadas a los refugiados en el hemisferio norte, la aplicabilidad de estos hallazgos en el contexto del SUS y en otros entornos de América Latina exige un análisis minucioso. A diferencia de los sistemas basados en seguros o de centros de recepción aislados, la atención en Brasil debe implementarse dentro de la red universal de la APS, velando por que no se fragmente la atención. Por lo tanto, las prácticas de acogida en territorios fronterizos, como el de Roraima, deben centrarse en el fortalecimiento de las Unidades Básicas de Salud como fuentes habituales de atención, integrando la vigilancia epidemiológica con la sensibilidad cultural necesaria para atender a las familias venezolanas.

## CONCLUSIÓN

La presente revisión de alcance ha mapeado las prácticas asistenciales de acogida dirigidas a los niños migrantes en la APS, poniendo de manifiesto que la acogida se ha llevado a cabo, predominantemente, mediante acciones clínicas y de seguimiento, medidas preventivas y la coordinación dentro de la red de servicios.

Los hallazgos refuerzan el papel central de la APS como puerta de entrada preferencial para la atención infantil y como coordinadora de la red de atención, lo que pone de manifiesto que la acogida es una tecnología relacional y organizacional fundamental para garantizar el acceso y la continuidad de la atención al niño migrante. La articulación intersectorial con los servicios de asistencia social, educación y protección social fue la categoría menos descrita en los estudios, lo que indica debilidades en la coordinación de la atención y en la implementación de políticas públicas integradas.

Por lo tanto, existe una clara necesidad de estudios futuros que aborden estrategias de acogida culturalmente competentes, intervenciones intersectoriales estructuradas y la evaluación de la efectividad de las prácticas asistenciales dirigidas a los niños migrantes en la APS.

De este modo, se recomienda el desarrollo de investigaciones futuras que profundicen en la comprensión de las necesidades de esta población y respalden la formulación de políticas y prácticas de atención más integradas, equitativas y centradas en el niño y la familia.

## Data Availability

Todo el conjunto de datos que respalda los resultados de este estudio se publicó en el propio artículo.
